# Consensus land-cover mapping improves grassland classification in European mountain landscapes

**DOI:** 10.1038/s41598-026-39197-w

**Published:** 2026-02-10

**Authors:** Šimon Opravil, Matthias Baumann, Tomáš Goga, Hamid Afzali, Tobias Kuemmerle, Róbert Pazúr

**Affiliations:** 1 Institute of Geography, Slovak Academy of Sciences, Štefánikova 49, Bratislava, 814 73 Slovak Republic; 2 Geography Department, Humboldt-University Berlin, Unter den Linden 6, 10099 Berlin, Germany; 3 Swiss Federal Institute for Forest, Snow and Landscape Research WSL, Zürcherstrasse 111, Birmensdorf, 8903 Switzerland; 4 Integrated Research Institute on Transformations of Human-Environment Systems (IRI THESys), Humboldt-University Berlin, Unter den Linden 6, 10099 Berlin, Germany

**Keywords:** Land-cover mapping, Consensus approach, Grasslands, Alps and Carpathians, Earth observation, Ecology, Ecology, Environmental sciences

## Abstract

Accurate land-cover information is essential for biodiversity monitoring, yet existing 10-m global and continental land-cover datasets vary in accuracy and thematic consistency, particularly for grasslands in complex mountain environments. We assessed six state-of-the-art land-cover products (Dynamic World, ESA WorldCover, Esri Land Cover, Corine Land Cover+ Backbone, ELC10, S2GLC) across the Alps and Carpathians and developed three consensus maps using weighted voting, accuracy-confusion weighting, and an accuracy-weighted Random Forest ensemble. All datasets were validated against an independent set of expert-interpreted reference samples. Individual products showed large discrepancies in grassland extent, elevation distribution, and landscape structure. Global datasets (Dynamic World, Esri Land Cover) underestimated grassland extent, whereas ESA WorldCover and Corine Land Cover+ reported higher proportions. Consensus approaches substantially reduced these inconsistencies. The Random Forest ensemble achieved the highest accuracy (overall 90–92%), outperforming individual datasets and improving both user’s and producer’s accuracies for grassland (> 84%). Consensus datasets also better captured expected elevation and slope gradients, producing more spatially coherent and ecologically realistic grassland patterns. By integrating multiple land-cover sources, consensus approaches effectively mitigated dataset-specific biases and increased the reliability of grassland mapping in heterogeneous mountain systems. Consequently, consensus land-cover products provide a robust and ecologically meaningful alternative to single-source datasets for environmental assessments in complex mountain regions.

**Supplementary Information:**

The online version contains supplementary material available at 10.1038/s41598-026-39197-w.

## Introduction

Land cover mapping is key to biodiversity monitoring and environmental reporting because it provides spatially explicit indicators of ecosystem state and change. Land-cover datasets are used to describe broad patterns of land use, but also as critical predictors in species distribution models and ecosystem service assessments^1–3^. By linking mapped habitats to landscape metrics, land-cover information can be translated into indicators of fragmentation, habitat quality, and ecological connectivity^4–7^. However, the validity of land-cover derived indicators depends directly on the accuracy and thematic consistency of the maps^8,9^.

This challenge is particularly evident in mountain ecosystems, which are among the most biodiverse regions, such as Alpine and Carpathian grasslands. These areas are considered biodiversity hotspots that have been shaped by centuries of traditional management, and are also linked to steep environmental gradients, complex climatic mosaics, and topographic heterogeneity, which together require land-cover maps that reliably capture grassland extent, distribution, and landscape structure^10–13^.

Over the past decades, numerous land-cover datasets have been developed at regional to global scales. These products vary considerably in terms of thematic detail, spatial resolution, and temporal coverage. Improvements in spatial and temporal resolution have not always been matched by corresponding increases in thematic accuracy. Comparative studies have shown that new 10-m global land-cover products differ sharply in how they represent major classes. For example, recent assessments indicate that global datasets tend to underestimate the extent of grasslands. Dynamic World and Esri Land Cover report only 9–12% of the Alps and Carpathians as grassland, while continental products such as Corine Land Cover + and ESA WorldCover map 21–24%, and ELC10 and S2GLC yield intermediate values (11–21%)^14,15^. These inconsistencies leave users without clear guidance on which dataset is most reliable for ecological applications.

On the other hand, this growing availability of concurrent land-cover datasets has opened opportunities to create consensus maps that combine multiple sources to exploit their strengths and offset individual weaknesses. Early approaches relied on majority voting or fuzzy logic, assigning degrees of class membership^14–16^. More sophisticated methods incorporate uncertainty and class-specific accuracy through the Dempster–Shafer theory or multi-criteria integration^17,18^, while conditional-probability models weight class assignments by reported accuracies^19^. Beyond these rule-based techniques, ensemble machine learning offers a flexible alternative, combining diverse classifiers to enhance predictive stability and reduce misclassification^20,21^. Despite its success in other domains, ensemble learning remains under-explored in land-cover harmonization, leaving open whether it can deliver more coherent and ecologically realistic consensus maps.

In this study, we focus on the Alps and the Carpathians to examine how grasslands are mapped across a suite of widely used land cover products and whether consensus approaches can improve their reliability. Specifically, we integrate multiple datasets containing grassland classes to generate consensus maps and evaluate their performance relative to individual products. In addition, we assess how these datasets capture grassland patterns along environmental gradients of elevation, slope, and aspect, and examine the implications of their differences for landscape metrics. Our research is guided by three questions:

1. Does a consensus approach improve the accuracy of grassland mapping compared to individual land cover datasets?

2. How consistently do different datasets map grasslands across variable terrain gradients?

3. What is the influence of the different land cover datasets on the overall landscape characteristics as defined by the landscape metrics?

## Data and methods

### Study areas

**Fig. 1:**
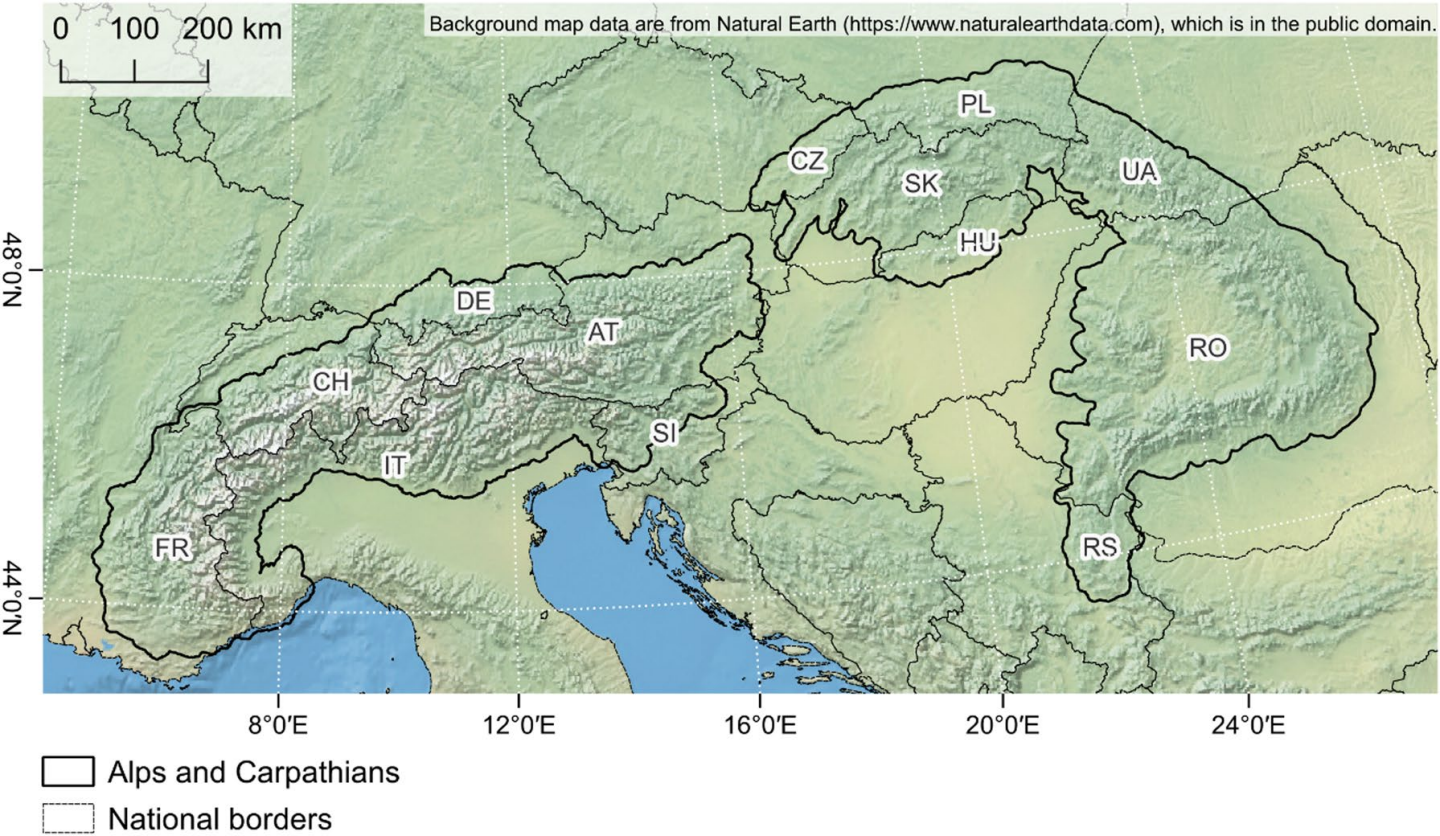
Study areas within Europe: the Alps and the Carpathians. Administrative boundaries and background map data are based on Natural Earth (https://www.naturalearthdata.com/). The figure was produced using QGIS 3.40 (https://qgis.org).

The Alps and Carpathians are the two largest European mountain ranges, differing in environmental and socio-economic settings that shape grassland ecosystems. The Alps span seven countries with temperate to alpine climates and steep precipitation gradients, while the Carpathians stretch across eight countries with more continental conditions (Fig. 1). Both regions contain mosaics of semi-natural grasslands managed by grazing and mowing but differ in management intensity and land-use history, from intensively used Alpine valleys to more extensive Carpathian systems shaped by collectivisation and post-socialist transitions.

### Data

#### Base land cover datasets

We integrated six state-of-the-art land-cover datasets to build a consensus land-cover map for the Alps and Carpathians: Dynamic World^22^ (DW), ESA WorldCover^23^ (ESA WC), Esri Land Cover^24^ (ESRI LC), Corine Land Cover+ Backbone (CLC+), ELC10^27^, and S2GLC^25^. All datasets provide 10 m spatial resolution and are derived from Sentinel-1 and Sentinel-2 observations, ensuring a consistent remote-sensing foundation.

The selected products vary in scope and purpose, ranging from global (DW, ESA WC, ESRI LC) to continental (CLC+, ELC10, S2GLC). All employ extensive training data, advanced modelling approaches, and comparable thematic classification schemes (Tables 1 and 2). While CLC + and S2G LC do not fully cover parts of Ukraine, each dataset contributes unique methodological strengths and weaknesses that together enable robust regional harmonization. (Supplementary S1).

#### Base datasets preprocessing

We harmonised the land-cover datasets by clipping them to the region of interest and standardising land-cover classes to a common classification scheme (Table 2). We used the top-level land-cover classification nomenclature from the Land Use/Cover Area frame statistical Survey (LUCAS)^26^, which includes artificial land, arable land, forest, shrubs, grassland, bare land, water, and wetland. Additionally, we included a class for glaciers and permanent snow based on LUCAS subclass G50. The ELC10 and DW datasets already followed this classification scheme, and we standardized the classification schemes of the other datasets based on Table 2. Additionally, we processed the DW datasets by applying the mode function to the multi-year dataset (2018–2019). This multi-year aggregation approach accounts for the seasonal variability inherent in DW’s temporal coverage, allowing for a more stable representation of persistent land-cover patterns. Consequently, it enhances the identification of long-term trends useful for distinguishing between cropland (both active and fallow) and grassland^27^.

**Table 1:** Overview of base land-cover maps containing grassland classes used in our study. MMU refers to the minimal mapping unit.

Dataset name	Abbreviation	Generated by	MMU (m^2^)	Years used	Coverage
Corine Land Cover+ Backbone	CLC+	Copernicus Land Monitoring Service	100	2018	Europe
Dynamic World	DW	Brown et al. (2022)	250	2018–2020	global
pan-European land cover map	ELC10	Venter & Sydenham (2021)	100	2018	Europe
WordCover ESA	ESA WC	Zanaga et al. (2020)	100	2020	global
Esri Land Cover	ESRI LC	Karra et al. (2021)	250	2020	global
Sentinel-2 Global Land Cover	S2GLC	Malinowski et al. (2020)	100	2017	Europe

#### Sampling validation data

We followed best practices for accuracy assessment^28^. Harmonised land-cover classes from the base maps defined sampling strata for validation pixels in each mountain region. To avoid bias, we used a single validation dataset stratified by an external land-cover map^29^, independent of the evaluated products but using identical class definitions. We targeted an overall accuracy standard error below 0.01, expecting higher user accuracy for artificial land, cropland, woodland, and water, and lower user accuracy for shrubland, grassland, bare land, wetland, and snow (Supplementary S2). Each land-cover class included at least 70 validation pixels, yielding 1,460 points in the Alps and 1,410 in the Carpathians (Supplementary S2). Three experts visually interpreted each point using 2018 high-resolution Google Earth and Sentinel-2 imagery. The final class was assigned by majority agreement or by a single expert when confident in their interpretation.

**Table 2:** Legend harmonisation of base LC datasets.

Abbr.	Built	Crop	Forest	Shrub	Grass	Bare	Water	Wet	Snow
Class Num.	1	2	3	4	5	6	7	8	9
LUCAS	Artificial land	Cropland	Woodland	Shrubland	Grassland	Bareland	Water	Wetlands	Snow
CLC+	Sealed	Periodically herbaceous	Woody	Low-growing woody plants	Permanent herbaceous	Non and sparsely-vegetated, Lichens and mosses	Water		Snow and ice
DW	Built Area	Crops	Trees	Scrub/Shrub	Grass	Bare Ground	Water	Flooded Vegetation	Snow/Ice
ELC10	Artificial land	Cropland	Woodland	Shrubland	Grassland	Bareland	Water	Wetlands	
ESA WC	Built-up	Cropland	Tree cover	Shrubland	Grassland	Bare / sparse vegetation, Moss and lichen	Permanent water bodies	Herbaceous wetland	Snow and Ice
Esri	Built Area	Crops	Trees	Scrub/Shrub	Grass	Bare Ground	Water	Flooded Vegetation	Snow/Ice
S2GLC	Artificial surfaces	Cultivated areas	Broadleaf, Coniferous tree cover	Moors and heathland	Herbaceous vegetation	Natural material surfaces	Water bodies	Marshes, Peatbogs	Permanent snow cover

### Evaluation and integration of land-cover datasets

#### Accuracy assessment

We validated each of the used land-cover datasets and the consensus dataset using the independent validation sample described in Sect. 2.2.3. For each class in each map, we computed area-weighted estimates for user accuracy, producer accuracy, overall accuracy, and the F1-score following Stehman and Foody (2019)^30^. Standard errors for these metrics were estimated using the equations provided by Stehman & Foody (2019, Eqs. 12–18)^30^.

#### Consensus approaches

We tested three consensus mapping approaches to integrate multiple land-cover datasets. First, we implemented a weighted voting approach (Con_WV), where each pixel was assigned the class with the highest sum of weights, with the weights being the F1 scores for each class across all input datasets. To enhance this weighted voting approach, we introduced an accuracy-confusion weighing method (Con_AccCo), adapted from Tuanmu & Jetz (2014)^19^. This method calculates a consensus probability for each class by summing correct classification probabilities (the diagonal elements of the error matrices) while subtracting misclassification probabilities (the off-diagonal elements), thereby emphasizing classes that are more reliably detected across datasets. Finally, we developed a Random Forest model (Con_RF), which integrates multiple land-cover datasets by encoding their class-specific performance into the feature space. Specifically, class-wise F1-scores are used as predictor variables where for each pixel, only the F1-score corresponding to the class assigned by a given dataset is retained, while all other class values are set to zero. This allows us to implicitly weigh the contribution of each dataset according to its observed class reliability during training. Training data were derived from LUCAS Harmonised^26^ and LUCAS Copernicus^31^ and preprocessed to remove ambiguous or low-accuracy points. The Random Forest classifier (ee.Classifier.smileRandomForest) was trained in Google Earth Engine with 500 trees and default parameters. For a more detailed description of these approaches, refer to Supplementary Material S1.

### Comparison of grasslands across the Alps and Carpathians

We first quantified the total extent of grasslands, defined as the area of all grassland pixels, in the Alps, in the Carpathians, and separately for each country within these mountain ranges. We then examined how this extent varied along environmental gradients. For every pixel classified as grassland, we extracted elevation, slope, and aspect from the Copernicus 30-m global DEM, resampled to 10 m using nearest-neighbour resampling.

To assess how grassland landscape structure differs among datasets, we compared the composition and configuration of landscapes in the Alps and Carpathians using a set of landscape metrics. Landscapes were selected by generating a regular point grid at 5-km spacing and drawing a circular buffer of 2500 m around each point. Within each buffer, we calculated metrics that describe grassland patch patterns in relation to the area and shape of grassland patches. The metrics include mean grassland patch area, total grassland area, patch fractal dimension (PAFRAC), and edge density.

## Results

### Comparison of individual land-cover maps and consensus map quality

**Fig. 2:**
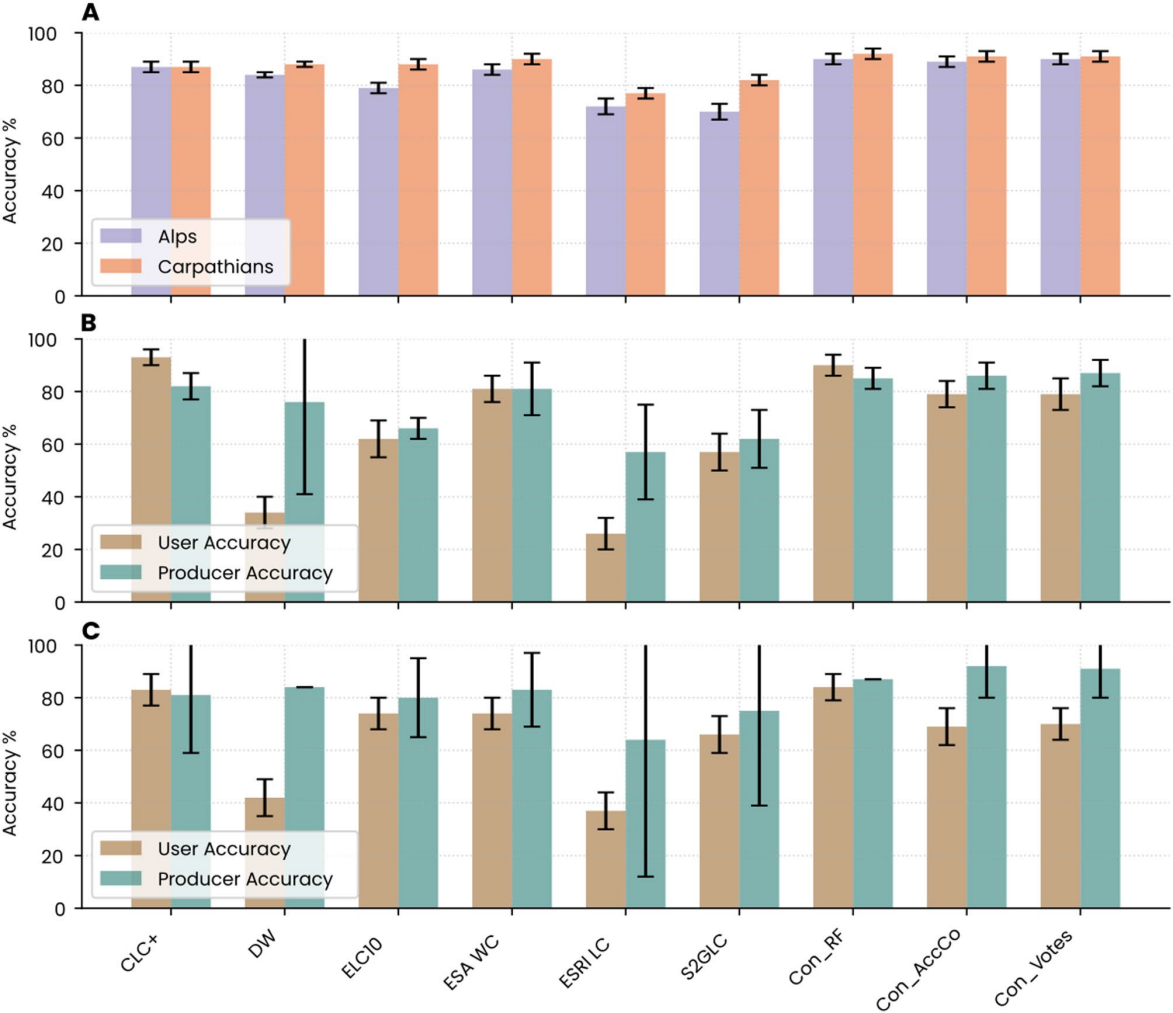
Accuracy assessment of the consensus (Con_RF) and individual land-cover datasets. (**A**) Overall accuracy for the Alps and Carpathians. (**B**) User and producer accuracy for the grassland class in the Alps. (**C**) User and producer accuracy for the grassland class in the Carpathians. Error bars represent 95% confidence intervals estimated from the corresponding error matrices.

The accuracy of individual base land-cover datasets varied considerably, reflecting differences in classification methods and input data (Fig. 2). In the Alps, overall accuracy ranged from 70% to 87%, with ESA WC (86%) and CLC+ (87%) performing best, while S2GLC (70%) and ESRI LC (82%) had the lowest accuracy among the evaluated datasets (Fig. 2A). Accuracy followed a similar pattern in the Carpathians, with ESA WC reaching 90% and CLC + and ELC10 (exceeding 87%). Both Con_WV and Con_AccCo improved overall classification accuracy relative to individual datasets, but Con_RF consistently achieved the highest accuracy, with an overall accuracy of 90% in the Alps and 92% in the Carpathians (Fig. 2A).

For grassland areas, the greatest improvement in the accuracy for this class was observed in Con_RF, where both user accuracy and producer accuracy exceeded 84% in the Alps and Carpathians (Fig. 2B, C). Con_RF and CLC+ emerged as the top-performing maps for grassland classification, with both datasets achieving user and producer accuracies above 80%. In contrast, Con_AccCo and Con_Votes demonstrated slightly higher omission errors by underestimating the grassland areas, which led to reduced user accuracy. Among the original datasets, only CLC+ outperformed Con_RF in terms of user accuracy in the Alps. However, when considering producer accuracy, both datasets showed very similar performance (0.872 and 0.874 F1-scores for CLC + and Con_RF, respectively).

**Fig. 3:**
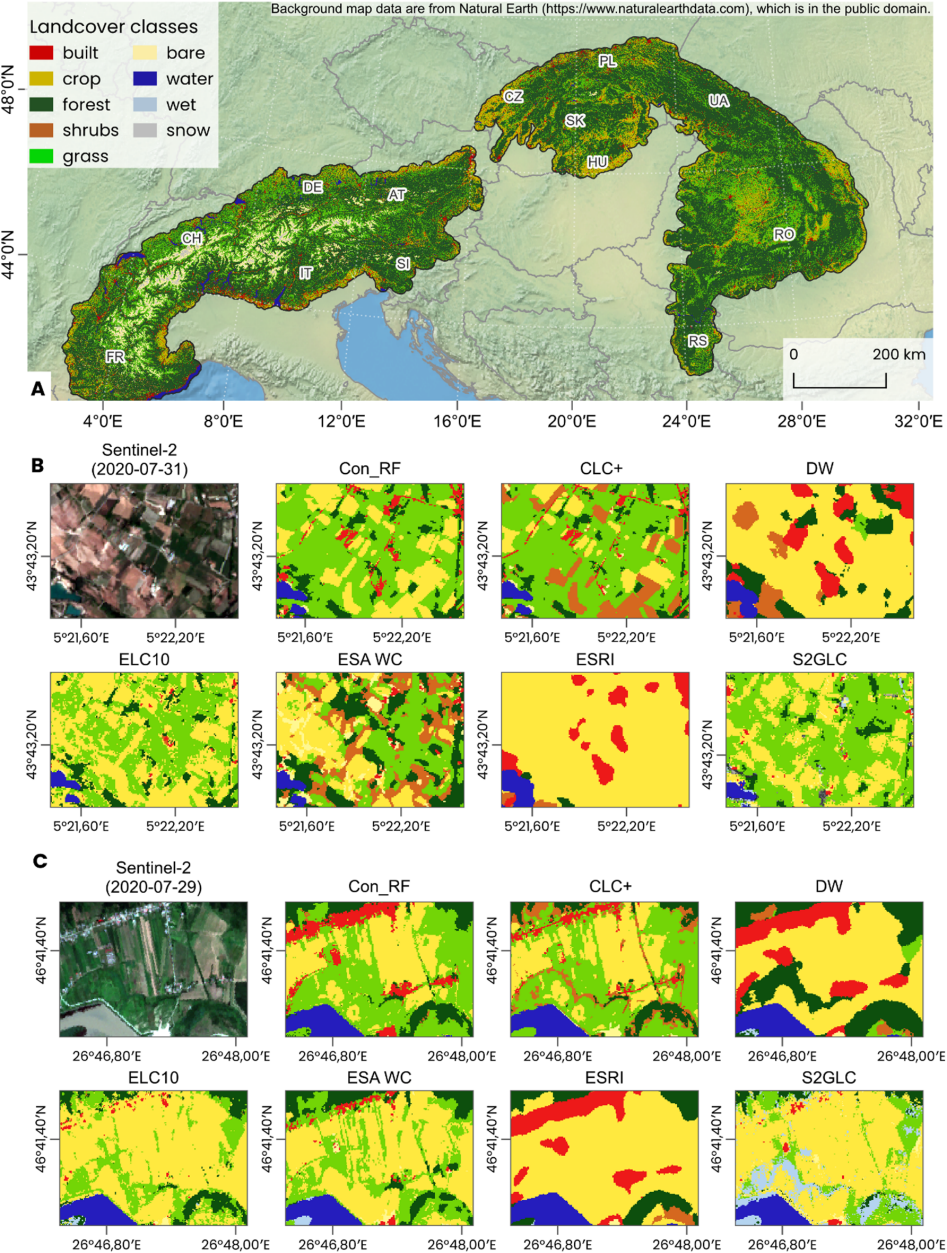
Comparison of the Con_RF consensus dataset with other land-cover datasets across the Alpine and Carpathian regions. (**A**) Con_RF land-cover map covering the Alps and Carpathians (**B**) Detailed example from the Alps (France) and (**C**) from the Carpathians (Romania). Administrative boundaries and background map data are based on Natural Earth (https://www.naturalearthdata.com/). Background imagery in panels (**B**) and (**C**) consists of Sentinel-2 Level-2 A true colour composites (Copernicus Sentinel-2). The figure was produced using QGIS 3.40 (https://qgis.org).

The visual comparison of differences is illustrated in Fig. 3, which contrasts the Con_RF consensus map with individual land-cover products across representative Alpine and Carpathian landscapes. Also, it is evident, how DW and ESRI LC underrepresent area of grasslands, compared to other land-cover datasets. In addition, differences in class aggregation and fragmentation between datasets are evident, supporting the interpretation of subsequent analyses.

### Inter-dataset differences in grassland extent and distribution

**Fig. 4:**
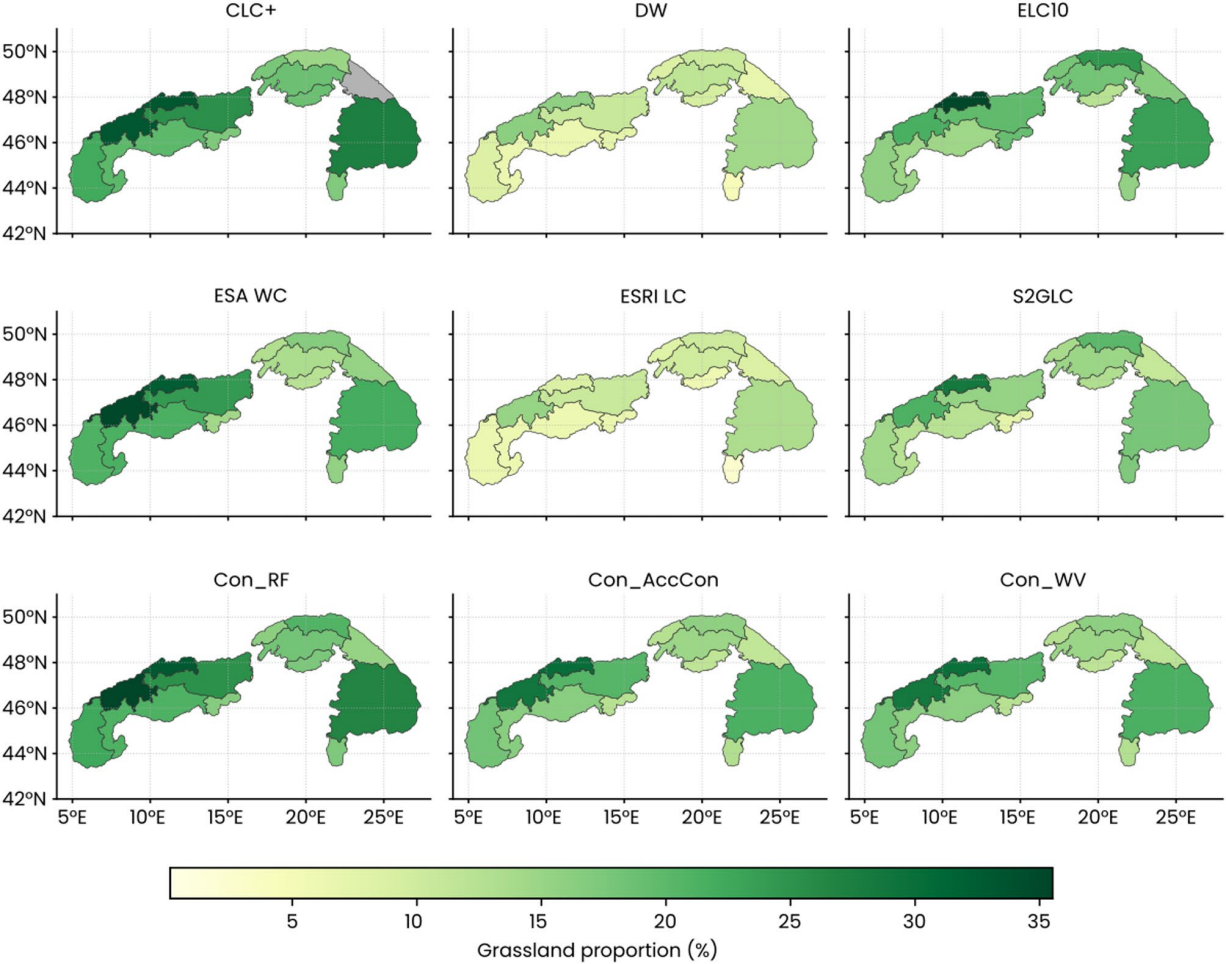
Country-level proportions of grasslands within the Alps and the Carpathians derived from different land-cover datasets. Grey color (CLC+) represents no data.

The comparison of land-cover datasets revealed clear discrepancies in mapped proportions of grasslands across countries in the Alps and Carpathians (Fig. 4). DW and ESRI LC consistently reported the lowest grassland coverage, whereas ESA WC and CLC+ showed the highest proportions. ELC10 produced intermediate values overall, but substantially higher grassland shares in the northwestern Alps (Germany) compared to all other products. Consensus products generally reduced low and high proportions but still differed in magnitude. Con_RF indicated higher grassland proportions than Con_AccCo and Con_WV, aligning more closely with ESA WC.

**Fig. 5:**
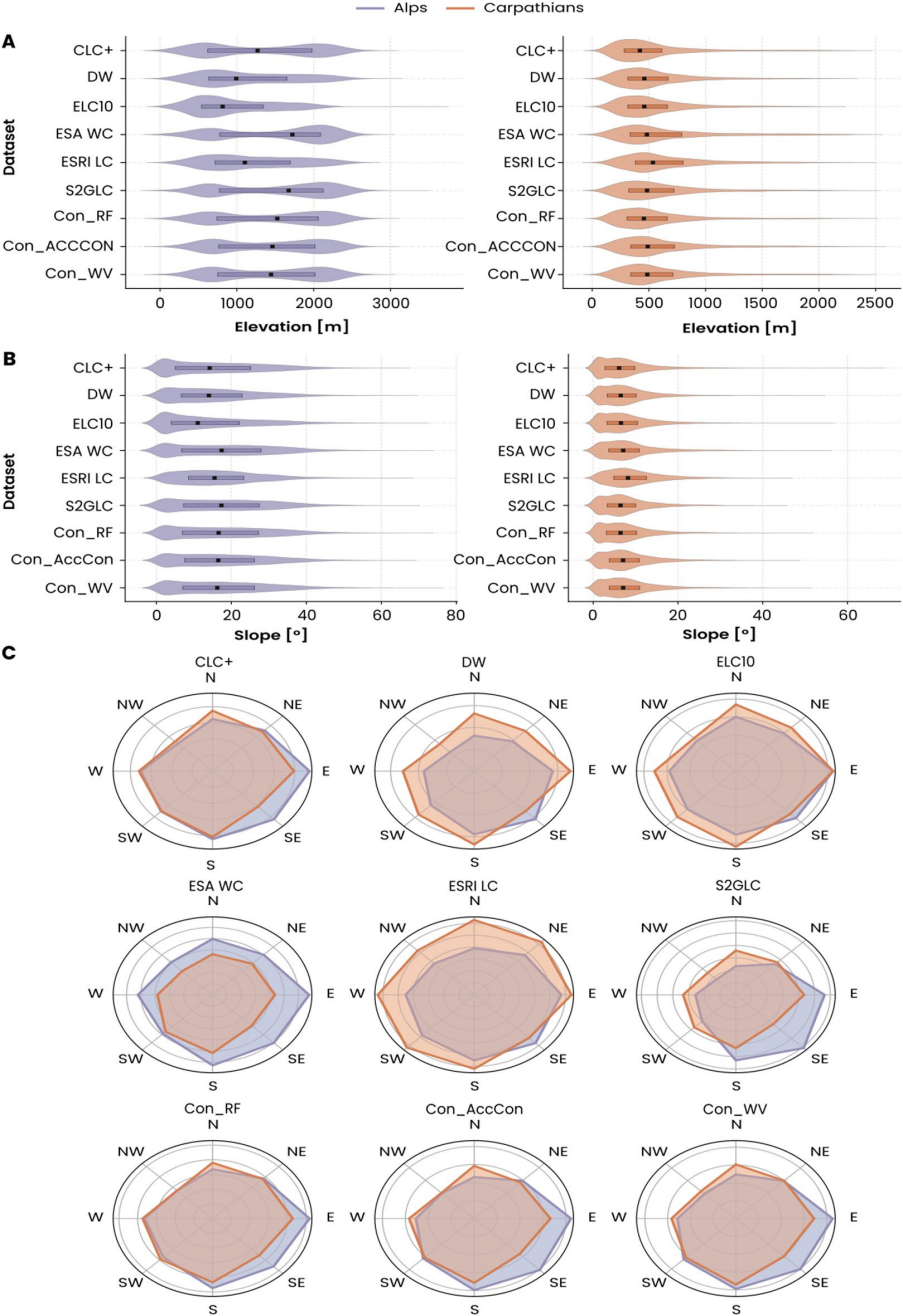
Terrain distribution of grasslands across compared land-cover datasets in the Alps and Carpathians. (**A**) Elevation, (**B**) slope, and (**C**) aspect.

The distribution of grasslands according to elevation, slope, and aspect gradients differs between individual map sources, which were more pronounced in the Alps than in the Carpathians (Fig. 5A). CLC+, ESA WC, and S2GLC covered the entire spectrum from secondary grasslands in lowlands to the alpine grasslands above 2,500 m a.s.l., while DW, ESRI LC and ELC10 mapped primarily lower elevation grasslands, with considerably lower representation in high mountain areas. Consensus products (Con_RF, Con_AccCo, Con_WV) produced more consistent spatial patterns but still varied in their depiction of high-elevation grasslands. Median values differed in the Alps, with a spread of 900 m between the lowest median (ELC10–810 m a.s.l.) and the highest ESA WC (1710 m a.s.l.). In the Carpathians, the differences between datasets were less pronounced in this respect, with all sources showing a distribution concentrated at lower elevations (with a median of 420 m a.s.l. for CLC + to 540 m a.s.l. for ESRI LC) and alpine locations almost absent.

Slope distribution and orientation (Fig. 5B) revealed less pronounced differences between the individual datasets as compared to elevation. In the Alps, CLC+, ELC10, and ESA WorldCover consistently mapped grasslands on gentler terrain, reflecting their tendency to classify grasslands on low slopes. In contrast, DW, ESRI LC, and S2GLC mapped grasslands to steeper slopes, especially in the upper mountain and subalpine zones.

In the Carpathians, most grasslands occurred on gentle terrain below 20°, corresponding to the extensive valley and plateau systems typical of this region. The narrower range of slopes compared to the Alps partly explains the reduced variability among datasets, as steep alpine meadows are almost absent in the Carpathian environment.

Differences in aspect distribution were the least pronounced yet still noticeable (Fig. 5C). In the Alps, CLC+, ELC10, and ESA WorldCover showed a preference for east to south-facing slopes, which corresponds to the climatic conditions of these exposures for productive grasslands. DW, ESRI LC and S2GLC captured similar orientations but placed greater emphasis on south and southeast orientations. In contrast, the Carpathian datasets exhibited a broader and more even distribution of aspects, reflecting the region’s gentler relief and lower topographic variability.

### Influence of land-cover dataset characteristics on grassland Spatial structure

**Fig. 6:**
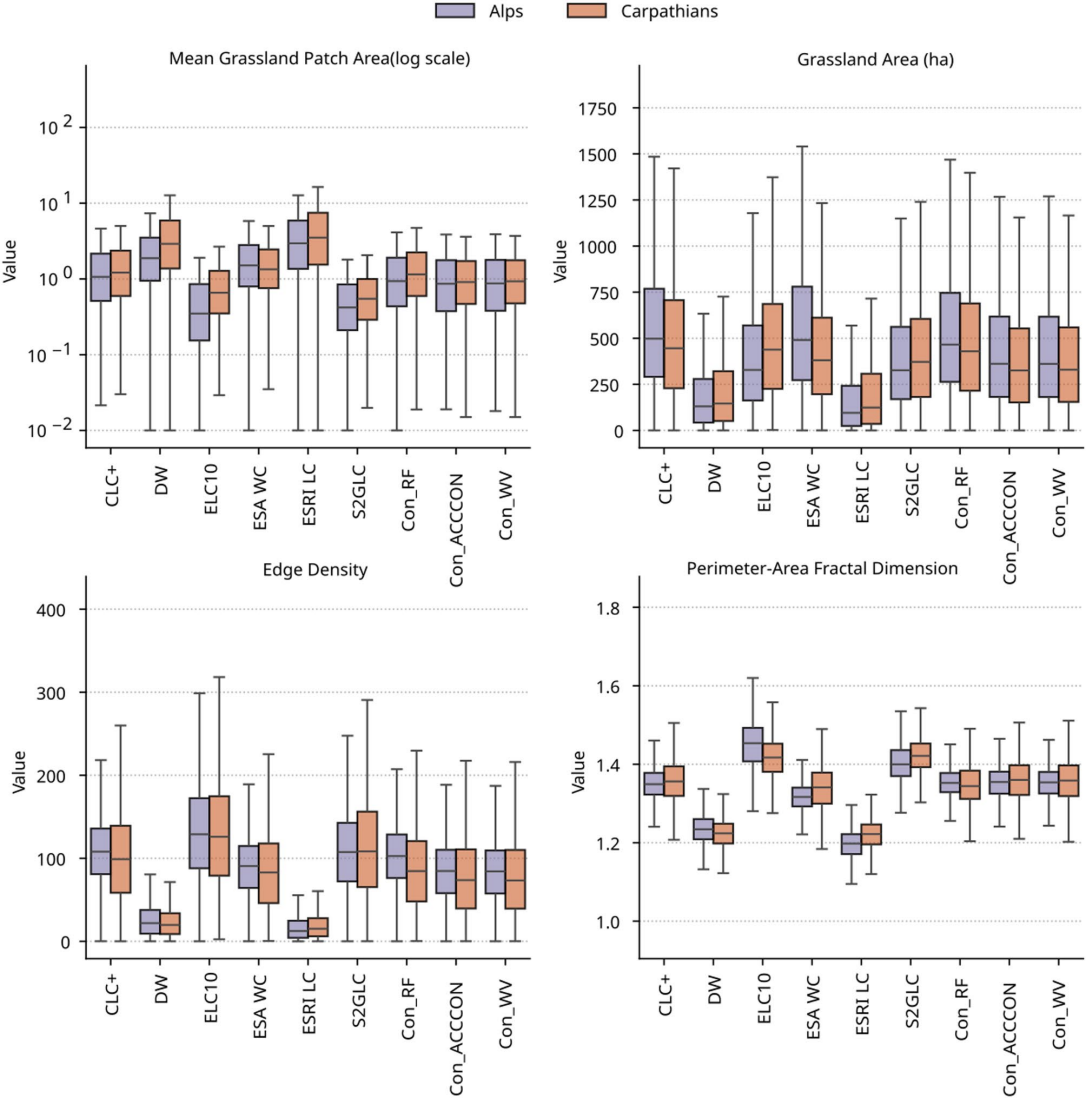
Comparison of landscape metrics for grasslands across datasets and regions. Boxplots show the distribution of mean grassland patch area (log scale), total grassland area, edge density, and perimeter–area fractal dimension.

The landscape metrics for grasslands across datasets and regions are summarized in Fig. 6. Landscape metrics reveal consistent structural patterns across datasets. ESRI LC and DW, with large mean patch areas, show low edge density and low shape complexity (PAFRAC). Accordingly, these datasets depict grassland classes as spatially aggregated, contiguous, and geometrically simple patches with limited but consolidated extent, as illustrated in Fig. 3B, C.

Conversely, ELC10 and S2GLC, characterized by small mean patch areas exhibit high edge density and elevated PAFRAC, reflecting fragmented, irregular, and heterogeneous landscapes. This spatial configuration is also evident in Fig. 3B and C, where grasslands and other land-cover classes appear highly dispersed and fine-grained. Their larger total class areas, concentrated mainly in the Carpathians, result from spatial dispersion rather than continuous coverage.

Intermediate configurations (e.g., ESA WC, CLC+, and consensus datasets) occupy a transitional position between these extremes. Moderate mean patch areas correspond with intermediate edge density and PAFRAC, reflecting balanced spatial aggregation and geometric complexity. Within the consensus datasets, Con_RF exhibits slightly larger mean patch and class areas, together with moderate edge density, suggesting that ensemble averaging mitigates fragmentation and produces a more cohesive yet regionally nuanced depiction of grassland structure.

## Discussion

Accurate and detailed land-cover mapping is essential for synthesizing knowledge of ecosystems across large areas. This need is especially acute in mountainous regions, where complex topography and diverse management histories generate mosaics of heterogeneous landscapes^10^. Within this study, we demonstrated that the land cover datasets provided contrasting mapping of grasslands in the Alps and Carpathians, with possible implications for ecological analyses and biodiversity monitoring. Furthermore, we showed that integrating multiple land-cover products reduces dataset-specific biases and yields a more reliable composite map^27,29,32^.

Our results confirm a central pattern reported for recent Sentinel-based land-cover datasets at 10 m resolution: while overall accuracy can be high, performance across individual classes remains uneven, and substantial differences persist between datasets^32^. In line with previous cross-comparisons, we observe that global datasets differ most in their ability to map grasslands, shrublands, and wetlands, while showing stronger agreement for water, tree cover, built-up areas, and crops^27^. Our assessment also confirms these class-specific discrepancies where DW and ESRI LC systematically underestimate the area of grasslands compared to ESA WC and CLC + in both mountain systems. Similar biases have been documented globally, with ESA WC tending to overestimate grasslands, Esri overestimating shrublands, and DW exaggerating snow/ice^14^. These systematic tendencies likely explain the under and overestimations of grassland areas that we found in the Alps and Carpathians.

In our comparison, the regional datasets (ELC10, CLC+, S2GLC) performed relatively well, echoing their reported pan-European accuracies^25,33^. However, our results show that the Alps and Carpathians pose particular challenges. The steep terrain, fine-scale mosaics, and spectral similarity between crops, grasslands, and shrubs exacerbate classification errors (Supplementary S4). Consequently, user and producer accuracies in our study deviated from continental assessments that documented persistent weaknesses in mapping herbaceous vegetation^34^.

Consensus approaches substantially improve the reliability of grassland mapping in complex mountain environments. The observed increase in their mapping accuracy highlights the ability of ensemble integration to produce a more dependable map of grassland cover. While regional datasets such as CLC + and ELC10 already performed relatively well, our results show that combining multiple sources reduces class-specific biases and produces depictions of grasslands that are both more robust and ecologically consistent. This supports previous calls for ensemble or weighted-evidence strategies to increase the reliability of land cover information^17,35,36^.

Nonetheless, consensus maps remain constrained by the quality and availability of the underlying datasets. For example, the lack of full coverage of the Carpathian Mountains in CLC + and the partial availability of S2GLC in Ukraine, limited their contribution within this part of mountain range. In addition, while our reference data followed best practices for class-based stratification, they were not explicitly stratified along elevation gradients, which restricts direct validation of dataset performance in high-elevation and alpine grasslands. Future research could address this by designing reference datasets that explicitly incorporate topographic gradients, particularly in alpine environments where grasslands are ecologically distinct and spectrally challenging. Moreover, harmonising the consensus map with the LUCAS nomenclature thematic detail leads to inconsistencies in wetland or snow classes across products. These compromises are in line with other harmonisation efforts, highlighting that consensus approaches can substantially reduce uncertainty, but cannot fully eliminate structural limitations inherent in current land-cover data sources^37,38^. Further progress will therefore depend on improved pan-European dataset coverage and denser, topographically stratified in situ observations in mountain regions.

The selection of land-cover datasets strongly influences the evaluation of environmental gradients and landscape metrics (Figs. 4 and 5). We documented that on grasslands, where their elevation distribution revealed strong divergences. Some datasets reproduced the expected bimodal pattern of secondary lowland and alpine grasslands in the Alps, while others mapped larger extents of grasslands in low elevations. Such bias in the datasets may underestimate the high-elevation grasslands, although this cannot be directly validated in this study, given that our reference data were not stratified by elevation. Even so, we found that in the Carpathians, differences between datasets were less pronounced, which is likely due to the location of grasslands at lower and mid elevations. Slope distributions showed narrower variation than elevation but still revealed potential biases. ELC10 and CLC+ emphasised gentler slopes, while DW and ESRI LC extended into steeper terrain. Aspect patterns were more subtle, with ESA WC and CLC+ depicting grasslands more evenly across orientations, while DW and Esri concentrated them on south- and east-facing slopes. Selected landscape metrics reinforced these distinctions, as DW and ESRI LC mapped grasslands as aggregated patches due to their minimum mapping unit area, whereas other datasets depicted finer, more fragmented mosaics. Consensus maps reconciled these extremes, producing more ecologically plausible patch structures and retaining regional contrasts between the Alps and Carpathians.

These improvements have important implications for downstream applications. Venter et al. (2023)^39^, for example, showed that Sentinel-based land-cover maps improved species distribution models for solitary bees in Norway compared with manually digitised maps, largely because products such as ELC10 and ESA WC, with smaller minimum mapping units, better resolved fine-scale habitat patches. Natsukawa et al. (2024)^40^ demonstrated that integrating fine-scale land cover data with topographic information substantially enhances habitat models, yielding significant insights for conservation. Our findings suggest that relying on a single dataset for similar broad-scale analyses may yield markedly different results, depending on the specific product chosen.

By integrating multiple land-cover datasets, our consensus map mitigated these distortions, preserving ecologically relevant gradients while reducing outliers from individual datasets. This suggests that consensus approaches can provide land-cover maps that are ecologically plausible, are methodologically robust, and are more suitable for downstream applications. These include biodiversity modelling^19,39^, habitat quality assessments, connectivity analyses, and quantification of land use intensity in grassland systems. In this context, ensemble products also improve the comparability of studies that rely on land cover as a foundational variable. Relying on a single land-cover dataset risks systematic bias, whereas consensus products enhance the comparability and credibility of evidence. In this sense, ensemble approaches improve the practical utility of land cover information in heterogeneous mountain landscapes.

## Conclusion

Accurate land cover maps are fundamental for broad-scale monitoring of the earth-systems particularly in heterogeneous mountain systems. Thanks to recent technical availabilities, there are multiple datasets that are candidates to reach sufficient accuracy. Our analysis revealed that state-of-the-art land-cover datasets, although derived from similar satellite sources, yield substantially different representations of grasslands in the Alps and Carpathians, which in turn affects their extent and spatial configuration. These discrepancies varied across gradients of elevation, slope, and aspect. By integrating multiple datasets through ensemble and weighted consensus approaches, we demonstrated a reduction of such discrepancies. The Random Forest consensus map achieved the highest overall and class-specific accuracies, outperforming individual datasets and producing an ecologically coherent map of grasslands in both mountain regions. These improvements arise from integrating the complementary strengths of multiple land-cover products, which reduces dataset-specific biases and enhances robustness in complex mountain terrain. Consequently, integrative multi-dataset approaches offer a robust pathway toward producing more dependable land-cover information in mountainous regions.

Beyond accuracy gains, the consensus approach preserved key ecological gradients and generated more realistic spatial patterns of grassland distribution and fragmentation. Such qualitative improvements may be particularly valuable for downstream applications that rely on land-cover inputs. While it may directly impact the results of the spatial-based studies, such as habitat modelling, landscape connectivity analyses, and biodiversity monitoring, it may also indirectly influence the policy documents that shape the future biodiversity strategies.

## Supplementary Information

Below is the link to the electronic supplementary material.

Supplementary Material 1

## Data Availability

Consensus datasets are openly available in the Zenodo repository at (10.5281/zenodo.13823832). The code used for data processing, analysis, and all other datasets stored in Google Earth Engine is publicly accessible in the GitHub repository at https:/github.com/simonopravil/MAC-Land.
