# Supplementary material for: Consensus land-cover mapping improves grassland classification in European mountain landscapes
Source: Sci Rep. 2026 Feb 10;16:8077. doi: 10.1038/s41598-026-39197-w (PMC12960803; doi:10.1038/s41598-026-39197-w)
Supplement: Supplementary file 1 — Supplementary Material 1 [file 41598_2026_39197_MOESM1_ESM.docx]

Opravil, S., Baumann, M., Goga, T., Afzali, H., Kuemmerle, T., Pazur, R. 2025. Consensus land-cover mapping improves grassland classification in European mountain landscapes

**Table of content**

[Supplementary material S1: Text](#_o9khhb3ee10j)

[Text A.1: Detailed description of study areas](#_srx4njt1sbnr)

[Text A.2: Comprehensive description of the used land-cover datasets](#_x9ql8fy13xxl)

[Text A.3: Comprehensive description of consensus land-cover map approaches](#_ke79teeh1ske)

Random Forest

[Weighted votes](#_pnya9qyfljiu)

[Accuracy-Confusion](#_4d34og8)

[Supplementary material S2: Tables](#_diqf40cb5tl3)

[Table S.2: McNemar Test Results for Overall Accuracy Comparison of Land Cover Maps: Confidence Intervals, Chi-Square, and p-Values](#_w5mfngkftz6g)

[Supplementary material S3: Figures](#_dxq4gqn58pc9)

[Figure S.1. User and Producer accuracies for each dataset class, A - Alps, B - Carpathians](#_8qaq0vnmymre)

[Supplementary material S4: Error Matrices - Alps](#_ir8bg6njud6p)

[Table S2. Error matrix for Con_RF Alps, map area weights (Wi), user's accuracy (UA), producer's accuracy (PA) and overall accuracy (OAA) for non-adjusted and area-adjusted calculation with class-wise confidence intervals.](#_6vikro1oi6cc)

[Table S3. Error matrix for Con_AccCO Alps, Metrics as in Table S2.](#_y76g3uihu66f)

[Table S4. Error matrix for Con_WV Alps, Metrics as in Table S2.](#_pqf16qkxmwuc)

[Table S5. Error matrix for CLC+ Alps, Metrics as in Table S2.](#_olus8pb47wcy)

[Table S6. Error matrix for DW Alps, Metrics as in Table S2.](#_6ljeu5zflzmj)

[Table S8. Error matrix for ESA WC Alps, Metrics as in Table S2.](#_cw9aqwte01rw)

[Table S9. Error matrix for ESRI Alps, Metrics as in Table S2.](#_6ywhdbox09kq)

[Table S10. Error matrix for S2GLC Alps, Metrics as in Table S2.](#_wdvu3hujm2g6)

[Supplementary material: Error Matrices - Carpathians](#_43nbjjc50szv)

[Table S11. Error matrix for Con_RF Carpathians, Metrics as in Table S2.](#_k3fm7jvn7r63)

[Table S12. Error matrix for Con_AccCo Carpathians, Metrics as in Table S2.](#_6i7bxpjb6li8)

[Table S13. Error matrix for Con_WV Carpathians, Metrics as in Table S2.](#_4mumblt3tbev)

[Table S14. Error matrix for CLC+ Carpathians, Metrics as in Table S2.](#_7s1adghvti9e)

[Table S15. Error matrix for DW Carpathians, Metrics as in Table S2.](#_hw07yf1dmluc)

[Table S16. Error matrix for ELC10 Carpathians, Metrics as in Table S2.](#_h3qxsuczel0g)

[Table S17. Error matrix for ESA WC Carpathians, Metrics as in Table S2.](#_l4lddwvfalwc)

[Table S18. Error matrix for ESRI LC Carpathians, Metrics as in Table S2.](#_tbhm4invvb01)

[Table S19. Error matrix for S2GLC Carpathians, Metrics as in Table S2.](#_kgfcvl1ne841)

[References](#_kxnbutpa0vie)

# Supplementary material S1: Text

## Text A.1: Detailed description of study areas

The Alps and the Carpathians are the two major European mountain ranges, characterised by distinct environmental and socio-economic features that shape their grassland ecosystems.

The Alps span across various regions in seven countries, featuring a temperate climate in the foothills that gradually transitions to alpine conditions at higher elevations. Winters are snowy, and summers experience substantial precipitation. Alpine soils are often classified as Leptosols, which are shallow, acidic, and nutrient-poor. Alpine grasslands are a mix of natural and semi-natural meadows, intensively managed at lower elevations for hay production and grazing. Traditional practices have maintained high biodiversity, but recent agricultural intensification and land abandonment threaten habitat integrity[^1^](https://www.zotero.org/google-docs/?e4WgRU).

The Carpathians stretch across eight countries and are characterised by more continental climates, influenced by polar-continental air masses in winter and oceanic air masses in the other seasons. Precipitation decreases with distance from the Atlantic, creating drier intermontane depressions and on the lower southern slopes. Soils range from fertile Cambisols at lower elevations to Podzols and Leptosols at higher elevations. Overall, grasslands in the Carpathians are less intensively managed than those in the Alps. The landscape patterns in the Carpathians have been shaped by socialist-era collectivization and post-socialist transitions, which prioritized large-scale agricultural systems over diverse management approaches[^2^](https://www.zotero.org/google-docs/?8da08w). As a result, these grasslands typically exhibit larger patch sizes with uniform management structures alongside regional variations in abandonment and intensification.

## Text A.2: Comprehensive description of the used land-cover datasets

Dynamic World (DW) — Developed by Google and partners, DW is the first near real-time global land use/land cover product at 10 m resolution. It applies a fully convolutional neural network to Sentinel-2 L1C imagery and delivers per-pixel probability estimates for nine land cover classes (water, trees, grass, crops, shrub & scrub, flooded vegetation, built area, bare ground, snow/ice). Its continuous update stream allows flexible temporal aggregation and the ability to capture dynamic processes such as cropping cycles and seasonal flooding [^3^](https://www.zotero.org/google-docs/?HIr7o9).

Esri Land Cover (Esri LC) — Released in 2020, Esri’s global 10 m product is based on deep learning models applied to Sentinel-2 imagery, trained on billions of hand-annotated samples. It shares the same typology as DW but produces annual composites [^4^](https://www.zotero.org/google-docs/?Sr8tOH).

ESA WorldCover (ESA WC) — Produced by ESA in 2020 and 2021, WorldCover provides a global 10 m land cover map derived from both Sentinel-1 SAR and Sentinel-2 optical imagery. It was designed to improve global consistency and accuracy compared to previous ESA land-cover products [^5^](https://www.zotero.org/google-docs/?CdbwFc).

CLC+ Backbone (CLC+) — The Copernicus CLC+ Backbone is a pan-European product providing harmonised land cover at 10 m resolution for 2018. It is designed to underpin the next CORINE Land Cover generation and follows a highly standardised workflow integrating Sentinel-2 with existing Copernicus High-Resolution Layers. Its role is to ensure continuity of the long-standing CORINE programme.

ELC10 — The European LC map at 10 m resolution for 2018 was developed by Venter & Sydenham (2021)[^6^](https://www.zotero.org/google-docs/?SmgjdF) using Sentinel-1 and Sentinel-2 time series and LUCAS reference samples. The product achieved an overall accuracy of 90% across eight LUCAS-based classes. Importantly, ELC10 is fully open-source and reproducible within Google Earth Engine, enabling continuous updating and extending to local scales.

S2GLC — Produced by Malinowski et al. (2020)[^7^](https://www.zotero.org/google-docs/?Ns84mj), S2GLC is a continental-scale product for Europe at 10 m resolution for 2017. It employed an automated workflow combining multi-temporal Sentinel-2 with classification model trained on CORINE and High-Resolution Layers.

## Text A.3: Comprehensive description of consensus land-cover map approaches

### Random Forest

We selected the Random Forest classifier [^8^](https://www.zotero.org/google-docs/?5TxB4b) to create a con_RF land-cover map. Random Forest is widely used in the remote sensing community due to its excellent classification performance and fast processing capabilities. It handles large and noisy datasets effectively, captures non-linear relationships between explanatory and response variables, and is robust against overfitting [^8,9^](https://www.zotero.org/google-docs/?9KApoS).

Con_RF uses a Random Forest model to integrate base land-cover datasets using a matrix of F1 scores as input variables. This allows the model to consider the classification confidence of each dataset at the pixel level. The F1-score matrix is used as an input feature representation. Each pixel is described by a vector in which the mapped class of each dataset is associated with its class-specific F1-score, but only the F1-score corresponding to the class assigned by a given dataset is retained, while all other class values are set to zero. This enables the Random Forest to learn dataset reliability through decision tree splitting.

As training data, we used LUCAS *Harmonized* ([d’Andrimont et al., 2020](https://www.zotero.org/google-docs/?Nn6IY2); theoretical grid point data) and LUCAS *Copernicus* [(d’Andrimont et al., 2021](https://www.zotero.org/google-docs/?ib6SkR); polygon datasets), which are available in Google Earth Engine. We first filtered out the class F40 “other bareland” to preprocess the theoretical grid point data to avoid confusion between bare land and ploughed agricultural fields. We also filtered point data based on GPS accuracy, selecting only those with a location error (*gps_prec*) of less than 15 meters and a distance between the observation location and the LUCAS point (obs_dist) of less than 15 meters. The Copernicus polygon dataset was filtered using the “*copernicus_cleaned*” property. Finally, we converted the string representation of LUCAS LC1 classes to numeric values to ensure compatibility with classifier requirements. We trained the Random Forest classifier (*ee.Classifier.smileRandomForest*) in Google Earth Engine, using 500 trees and default settings for other model parameters.

### Weighted votes

As a second approach to creating a consensus land-cover (LC) dataset, we used a weighted voting method. In a standard majority vote, the most frequently occurring class in a pixel is selected as the final LC class. However, this method treats all datasets equally, even if some are less accurate for certain classes, leading to potential errors, especially in heterogeneous areas [^12^](https://www.zotero.org/google-docs/?Zfazpz). We applied a weighted vote to address this, using the same F1-score matrix as in the Random Forest model. The weights were summed based on dataset class accuracy, and the class with the highest weight was assigned as the pixel’s final LC class.

### Accuracy-Confusion

We also tested the modified Tuanmu & Jetz (2014, eq.1)[^13^](https://www.zotero.org/google-docs/?skv6eb) approach to create a consensus LC map. [Tuanmu & Jetz (2014)](https://www.zotero.org/google-docs/?fKDNI4) combined two LC maps with different spatial resolutions in the original approach. They calculated a consensus value for each class presented in a single pixel by weighting the probability of class i with its areal proportion in a pixel.

We did not apply the areal proportion weighting as we were working with datasets with the same spatial resolution. Instead, the method sums the correct classification probabilities (diagonal values from the error matrices) and subtracts each class's misclassification probabilities (off-diagonal values) for each dataset-class combination within a pixel. The method then averages these sums across the datasets to compute the final probability for each class (Eq. 1).

|  | $P(i) = \frac{1}{D} \sum_{d\epsilon D} E_{d}[i,i] -\sum_{i\neq j} E_{d}[i,j]$ | Eq. 1. |
| --- | --- | --- |

Where:
P(i) is the probability associated with class i,

D is a dataset count,

E_d_ represents the error matrix for a dataset,

E_d_[i,i] denotes the diagonal element of E_d_, representing the probability of correct classification for class i, E_d_[i,j] denotes the off-diagonal element of E_d_, where i≠j, represents the probability of misclassification from class i to class j.

# Supplementary material S2: Tables

*Table S.1. Allocation of validation samples following (Olofsson et al., 2014)*[^14^](https://www.zotero.org/google-docs/?FOjTIJ)*: Area weights per stratum (Wi), conjected user accuracies (UA), and stratum standard deviation (Si).*

|  | Alps | | | | | | Carpathians | | | | | |
| --- | --- | --- | --- | --- | --- | --- | --- | --- | --- | --- | --- | --- |
| Strata (i) | Wi | UA | Si | Prop. Alloc | Target | Results | Wi | UA | Si | Prop. Alloc | Target | Result |
| built | 0.04 | 0.84 | 0.37 | 52 | 70 | 81 | 0.03 | 0.84 | 0.37 | 35 | 70 | 74 |
| crop | 0.08 | 0.87 | 0.34 | 108 | 110 | 139 | 0.17 | 0.87 | 0.34 | 193 | 200 | 208 |
| forest | 0.48 | 0.90 | 0.30 | 629 | 630 | 646 | 0.56 | 0.90 | 0.30 | 637 | 640 | 675 |
| shrub | 0.06 | 0.66 | 0.47 | 81 | 80 | 59 | 0.02 | 0.66 | 0.47 | 25 | 70 | 80 |
| grass | 0.19 | 0.70 | 0.46 | 249 | 250 | 209 | 0.19 | 0.70 | 0.46 | 217 | 220 | 192 |
| bare | 0.08 | 0.78 | 0.41 | 106 | 110 | 158 | 0.00 | 0.78 | 0.41 | 4 | 70 | 60 |
| water | 0.02 | 0.96 | 0.20 | 29 | 70 | 72 | 0.01 | 0.96 | 0.20 | 7 | 70 | 77 |
| wet | 0.00 | 0.63 | 0.48 | 6 | 70 | 37 | 0.01 | 0.63 | 0.48 | 8 | 70 | 39 |
| snow | 0.03 | 0.67 | 0.47 | 42 | 70 | 57 | 0.00 | 0.00 | 0.00 | 0 | 0 | 0 |
| *n* |  |  |  | *1302* | *1460* | *1458* |  |  |  | *1126* | *1410* | *1405* |

##

#### Table S.2: McNemar Test Results for Overall Accuracy Comparison of Land Cover Maps: Confidence Intervals, Chi-Square, and p-Values

|  | | Alps | | | | Carpathians | | | |
| --- | --- | --- | --- | --- | --- | --- | --- | --- | --- |
| Consensus LC | Base LC | lower ci | upper ci | chi value | p value | lower ci | upper ci | chi value | p value |
| Con_RF | CLC+ | 0,01 | 0,04 | 12,89 | 0,000 | 0,05 | 0,10 | 45,25 | 0,000 |
|  | DW | 0,11 | 0,15 | 116,26 | 0,000 | 0,04 | 0,08 | 39,55 | 0,000 |
|  | ESA WC | 0,04 | 0,08 | 37,83 | 0,000 | 0,01 | 0,05 | 9,80 | 0,002 |
|  | ESRI LC | 0,13 | 0,18 | 143,91 | 0,000 | 0,09 | 0,14 | 89,16 | 0,000 |
|  | ELC 10 | 0,11 | 0,15 | 123,57 | 0,000 | 0,01 | 0,04 | 7,34 | 0,007 |
|  | S2GLC | 0,13 | 0,18 | 122,22 | 0,000 | 0,02 | 0,07 | 13,34 | 0,000 |
| Con_WV | CLC+ | 0,01 | 0,05 | 8,42 | 0,004 | 0,07 | 0,11 | 53,69 | 0,000 |
|  | DW | 0,11 | 0,15 | 141,02 | 0,000 | 0,05 | 0,09 | 65,79 | 0,000 |
|  | ESA WC | 0,04 | 0,08 | 48,83 | 0,000 | 0,03 | 0,06 | 26,47 | 0,000 |
|  | ESRI LC | 0,14 | 0,18 | 170,05 | 0,000 | 0,11 | 0,15 | 137,06 | 0,000 |
|  | ELC 10 | 0,11 | 0,15 | 117,12 | 0,000 | 0,02 | 0,06 | 16,43 | 0,000 |
|  | S2GLC | 0,13 | 0,18 | 133,20 | 0,000 | 0,03 | 0,08 | 24,48 | 1e-06 |
| Con_AccCo | CLC+ | 0,01 | 0,04 | 7,09 | 0,008 | 0,07 | 0,11 | 51,60 | 0,000 |
|  | DW | 0,11 | 0,15 | 139,32 | 0,000 | 0,05 | 0,09 | 66,67 | 0,000 |
|  | ESA WC | 0,04 | 0,07 | 47,04 | 0,000 | 0,03 | 0,06 | 25,41 | 0,000 |
|  | ESRI LC | 0,14 | 0,18 | 169,40 | 0,000 | 0,11 | 0,15 | 137,33 | 0,000 |
|  | ELC 10 | 0,11 | 0,15 | 113,06 | 0,000 | 0,02 | 0,06 | 15,54 | 0,000 |
|  | S2GLC | 0,13 | 0,18 | 130,68 | 0,000 | 0,03 | 0,08 | 23,40 | 1e-06 |

# Supplementary material S3: Figures


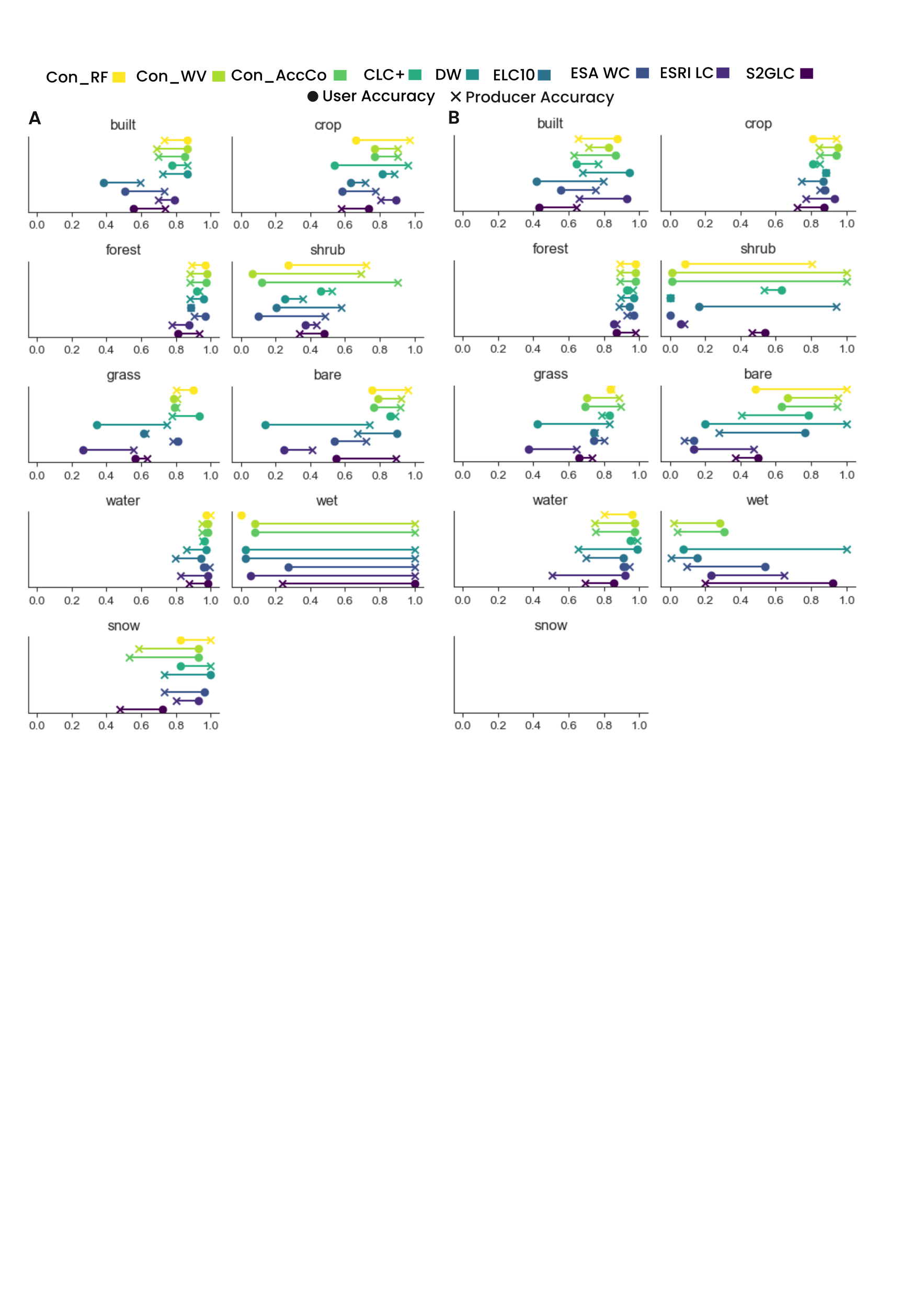


#### Figure S.1. User and Producer accuracies for each dataset class, A - Alps, B - Carpathians

# Supplementary material S4: Error Matrices - Alps

#### Table S2**.** Error matrix for Con_RF Alps, map area weights (Wi), user's accuracy (UA), producer's accuracy (PA) and overall accuracy (OAA) for non-adjusted and area-adjusted calculation with class-wise confidence intervals.

|  | **Reference** | | | | | | | | |  | **Map** | | | **Adjusted** | | | |
| --- | --- | --- | --- | --- | --- | --- | --- | --- | --- | --- | --- | --- | --- | --- | --- | --- | --- |
| **Con_RF** | built | crop | forest | shrub | grass | bare | water | wet | snow | Total | Wi | UA | PA | UA | UA ± | PA | PA ± |
| built | 70 | 0 | 1 | 2 | 7 | 1 | 0 | 0 | 0 | 81 | 0,03 | 0,86 | 0,83 | 0,86 | 0,08 | 0,80 | 0,06 |
| crop | 3 | 92 | 5 | 0 | 39 | 0 | 0 | 0 | 0 | 139 | 0,06 | 0,66 | 0,99 | 0,66 | 0,08 | 0,97 | 0,02 |
| forest | 5 | 0 | 627 | 3 | 11 | 0 | 0 | 0 | 0 | 646 | 0,49 | 0,97 | 0,90 | 0,97 | 0,01 | 0,94 | 0,18 |
| shrub | 0 | 0 | 30 | 16 | 13 | 0 | 0 | 0 | 0 | 59 | 0,06 | 0,27 | 0,59 | 0,27 | 0,11 | 0,34 | 0,03 |
| grass | 2 | 1 | 15 | 2 | 188 | 1 | 0 | 0 | 0 | 209 | 0,25 | 0,90 | 0,61 | 0,90 | 0,04 | 0,85 | 0,04 |
| bare | 4 | 0 | 10 | 2 | 23 | 119 | 0 | 0 | 0 | 158 | 0,08 | 0,75 | 0,91 | 0,75 | 0,07 | 0,95 | 0,00 |
| water | 0 | 0 | 1 | 0 | 1 | 0 | 70 | 0 | 0 | 72 | 0,02 | 0,97 | 0,95 | 0,97 | 0,04 | 1,00 | 0,00 |
| wet | 0 | 0 | 7 | 2 | 24 | 0 | 4 | 0 | 0 | 37 | 0,00 | 0,00 | 0,00 | 0,00 | 0,00 | 0,00 | 0,00 |
| snow | 0 | 0 | 0 | 0 | 0 | 10 | 0 | 0 | 47 | 57 | 0,01 | 1,00 | 1,00 | 0,82 | 0,10 | 1,00 | 0,00 |
| Total | 84 | 93 | 696 | 27 | 306 | 131 | 74 | 0 | 47 | 1458 | 1,00 | **OAA** | 0,84 | **OAA** | 0,90 | **OAA ±** | 0,02 |

#### Table S3**.** Error matrix for Con_AccCO Alps, Metrics as in Table S2.

|  | **Reference** | | | | | | | | |  | **Map** | | | **Adjusted** | | | |
| --- | --- | --- | --- | --- | --- | --- | --- | --- | --- | --- | --- | --- | --- | --- | --- | --- | --- |
| **Con_AccCo** | built | crop | forest | shrub | grass | bare | water | wet | snow | Total | Wi | UA | PA | UA | UA ± | PA | PA ± |
| built | 69 | 1 | 1 | 0 | 7 | 3 | 0 | 0 | 0 | 81 | 0,03 | 0,85 | 0,81 | 0,85 | 0,08 | 0,75 | 0,07 |
| crop | 3 | 107 | 5 | 0 | 24 | 0 | 0 | 0 | 0 | 139 | 0,06 | 0,77 | 0,94 | 0,77 | 0,07 | 0,89 | 0,02 |
| forest | 7 | 0 | 631 | 0 | 6 | 0 | 0 | 0 | 2 | 646 | 0,49 | 0,98 | 0,87 | 0,98 | 0,01 | 0,92 | 0,52 |
| shrub | 0 | 0 | 37 | 7 | 13 | 2 | 0 | 0 | 0 | 59 | 0,06 | 0,12 | 0,88 | 0,12 | 0,08 | 0,49 | 0,03 |
| grass | 3 | 6 | 30 | 0 | 166 | 3 | 1 | 0 | 0 | 209 | 0,25 | 0,79 | 0,67 | 0,79 | 0,05 | 0,86 | 0,05 |
| bare | 3 | 0 | 11 | 1 | 10 | 121 | 0 | 0 | 12 | 158 | 0,08 | 0,77 | 0,91 | 0,77 | 0,07 | 0,92 | 0,09 |
| water | 0 | 0 | 1 | 0 | 0 | 0 | 71 | 0 | 0 | 72 | 0,02 | 0,99 | 0,93 | 0,99 | 0,03 | 0,95 | 0,00 |
| wet | 0 | 0 | 7 | 0 | 23 | 0 | 4 | 3 | 0 | 37 | 0,00 | 0,08 | 1,00 | 0,08 | 0,09 | 1,00 | 0,13 |
| snow | 0 | 0 | 0 | 0 | 0 | 4 | 0 | 0 | 53 | 57 | 0,01 | 0,79 | 0,79 | 0,93 | 0,07 | 0,58 |  |
| Total | 85 | 114 | 723 | 8 | 249 | 133 | 76 | 3 | 67 | 1458 | 1,00 | **OAA** | 0,84 | **OAA** | 0,89 | **OAA ±** | 0,02 |

#### Table S4**.** Error matrix for Con_WV Alps, Metrics as in Table S2.

|  | **Reference** | | | | | | | | |  | **Map** | | | **Adjusted** | | | |
| --- | --- | --- | --- | --- | --- | --- | --- | --- | --- | --- | --- | --- | --- | --- | --- | --- | --- |
| **Con_WV** | built | crop | forest | shrub | grass | bare | water | wet | snow | Total | Wi | UA | PA | UA | UA ± | PA | PA ± |
| built | 70 | 1 | 1 | 0 | 6 | 3 | 0 | 0 | 0 | 81 | 0,03 | 0,86 | 0,80 | 0,86 | 0,08 | 0,74 | 0,07 |
| crop | 3 | 107 | 5 | 0 | 24 | 0 | 0 | 0 | 0 | 139 | 0,08 | 0,77 | 0,94 | 0,77 | 0,07 | 0,89 | 0,02 |
| forest | 7 | 0 | 633 | 1 | 5 | 0 | 0 | 0 | 0 | 646 | 0,52 | 0,98 | 0,87 | 0,98 | 0,01 | 0,92 | 0,20 |
| shrub | 0 | 0 | 38 | 4 | 15 | 2 | 0 | 0 | 0 | 59 | 0,05 | 0,07 | 0,67 | 0,07 | 0,06 | 0,14 | 0,03 |
| grass | 4 | 6 | 30 | 0 | 165 | 3 | 1 | 0 | 0 | 209 | 0,20 | 0,79 | 0,67 | 0,79 | 0,06 | 0,87 | 0,05 |
| bare | 3 | 0 | 11 | 1 | 9 | 125 | 0 | 0 | 9 | 158 | 0,09 | 0,79 | 0,91 | 0,79 | 0,06 | 0,93 | 0,09 |
| water | 0 | 0 | 1 | 0 | 0 | 0 | 71 | 0 | 0 | 72 | 0,02 | 0,99 | 0,93 | 0,99 | 0,03 | 0,95 | 0,00 |
| wet | 0 | 0 | 8 | 0 | 22 | 0 | 4 | 3 | 0 | 37 | 0,00 | 0,08 | 1,00 | 0,08 | 0,09 | 1,00 | 0,15 |
| snow | 0 | 0 | 0 | 0 | 0 | 4 | 0 | 0 | 53 | 57 | 0,01 | 0,85 | 0,85 | 0,93 | 0,07 | 0,62 |  |
| Total | 87 | 114 | 727 | 6 | 246 | 137 | 76 | 3 | 62 | 1458 | 1,00 | **OAA** | 0,84 | **OAA** | 0,90 | **OAA ±** | 0,02 |

#### Table S5**.** Error matrix for CLC+ Alps, Metrics as in Table S2.

|  | **Reference** | | | | | | | | |  | **Map** | | | **Adjusted** | | | |
| --- | --- | --- | --- | --- | --- | --- | --- | --- | --- | --- | --- | --- | --- | --- | --- | --- | --- |
| **CLC+ Backbone** | built | crop | forest | shrub | grass | bare | water | wet | snow | Total | Wi | UA | PA | UA | UA ± | PA | PA ± |
| built | 63 | 0 | 0 | 2 | 11 | 5 | 0 | 0 | 0 | 81 | 0,02 | 0,78 | 0,93 | 0,78 | 0,09 | 0,91 | 0,09 |
| crop | 1 | 75 | 3 | 21 | 39 | 0 | 0 | 0 | 0 | 139 | 0,06 | 0,54 | 0,99 | 0,54 | 0,08 | 0,95 | 0,02 |
| forest | 2 | 0 | 594 | 20 | 28 | 1 | 1 | 0 | 0 | 646 | 0,49 | 0,92 | 0,95 | 0,92 | 0,02 | 0,94 | 0,10 |
| shrub | 0 | 0 | 16 | 27 | 13 | 3 | 0 | 0 | 0 | 59 | 0,07 | 0,46 | 0,35 | 0,46 | 0,13 | 0,42 | 0,03 |
| grass | 0 | 1 | 9 | 2 | 195 | 2 | 0 | 0 | 0 | 209 | 0,24 | 0,93 | 0,59 | 0,93 | 0,03 | 0,82 | 0,05 |
| bare | 2 | 0 | 1 | 4 | 15 | 136 | 0 | 0 | 0 | 158 | 0,08 | 0,86 | 0,87 | 0,86 | 0,05 | 0,90 | 0,08 |
| water | 0 | 0 | 0 | 0 | 2 | 0 | 54 | 0 | 0 | 56 | 0,02 | 0,96 | 0,93 | 0,96 | 0,05 | 0,96 | 0,00 |
| wet | 0 | 0 | 3 | 2 | 29 | 0 | 3 | 0 | 0 | 37 | 0,00 | 0,00 | 0,00 | 0,00 | 0,00 | 0,00 | 0,00 |
| snow | 0 | 0 | 0 | 0 | 0 | 10 | 0 | 0 | 47 | 57 | 0,01 | 1,00 | 1,00 | 0,82 | 0,10 | 1,00 | 0,00 |
| Total | 68 | 76 | 626 | 78 | 332 | 157 | 58 | 0 | 47 | 1405 | 0,98 | **OAA** | 0,85 | **OAA** | 0,87 | **OAA ±** | 0,02 |

#### Table S6**.** Error matrix for DW Alps, Metrics as in Table S2.

|  | **Reference** | | | | | | | | |  | **Map** | | | **Adjusted** | | | |
| --- | --- | --- | --- | --- | --- | --- | --- | --- | --- | --- | --- | --- | --- | --- | --- | --- | --- |
| **DW** | built | crop | forest | shrub | grass | bare | water | wet | snow | Total | Wi | UA | PA | UA | UA ± | PA | PA ± |
| built | 70 | 2 | 4 | 3 | 1 | 1 | 0 | 0 | 0 | 81 | 0,07 | 0,86 | 0,65 | 0,86 | 0,08 | 0,72 | 0,05 |
| crop | 6 | 113 | 9 | 0 | 11 | 0 | 0 | 0 | 0 | 139 | 0,09 | 0,81 | 0,86 | 0,81 | 0,07 | 0,88 | 0,01 |
| forest | 12 | 0 | 621 | 1 | 2 | 0 | 1 | 0 | 9 | 646 | 0,54 | 0,96 | 0,82 | 0,96 | 0,01 | 0,89 | 0,14 |
| shrub | 1 | 0 | 39 | 15 | 1 | 0 | 0 | 0 | 3 | 59 | 0,04 | 0,25 | 0,21 | 0,25 | 0,11 | 0,44 | 0,10 |
| grass | 15 | 17 | 62 | 14 | 72 | 0 | 1 | 0 | 28 | 209 | 0,10 | 0,34 | 0,80 | 0,34 | 0,06 | 0,76 | 0,35 |
| bare | 3 | 0 | 13 | 20 | 1 | 22 | 5 | 0 | 94 | 158 | 0,02 | 0,14 | 0,92 | 0,14 | 0,05 | 0,77 | 0,08 |
| water | 1 | 0 | 0 | 1 | 0 | 0 | 70 | 0 | 0 | 72 | 0,02 | 0,97 | 0,86 | 0,97 | 0,04 | 0,92 | 0,00 |
| wet | 0 | 0 | 13 | 16 | 2 | 1 | 4 | 1 | 0 | 37 | 0,01 | 0,03 | 1,00 | 0,03 | 0,05 | 1,00 | 0,04 |
| snow | 0 | 0 | 0 | 0 | 0 | 0 | 0 | 0 | 57 | 57 | 0,10 | 0,30 | 0,30 | 1,00 | 0,00 | 0,76 |  |
| Total | 108 | 132 | 761 | 70 | 90 | 24 | 81 | 1 | 191 | 1458 | 1,00 | **OAA** | 0,71 | **OAA** | 0,84 | **OAA ±** | 0,01 |

*Table S7****.*** *Error matrix for ELC10 Alps, Metrics as in Table S2.*

|  | **Reference** | | | | | | | | |  | **Map** | | | **Adjusted** | | | |
| --- | --- | --- | --- | --- | --- | --- | --- | --- | --- | --- | --- | --- | --- | --- | --- | --- | --- |
| **ELC10** | built | crop | forest | shrub | grass | bare | water | wet | snow | Total | Wi | UA | PA | UA | UA ± | PA | PA ± |
| built | 31 | 15 | 5 | 0 | 23 | 6 | 1 | 0 | 0 | 81 | 0,01 | 0,38 | 0,89 | 0,38 | 0,11 | 0,67 | 0,09 |
| crop | 0 | 88 | 2 | 0 | 48 | 1 | 0 | 0 | 0 | 139 | 0,06 | 0,63 | 0,75 | 0,63 | 0,08 | 0,75 | 0,02 |
| forest | 3 | 3 | 571 | 10 | 38 | 16 | 5 | 0 | 0 | 646 | 0,42 | 0,88 | 0,92 | 0,88 | 0,02 | 0,94 | 0,12 |
| shrub | 0 | 1 | 20 | 12 | 11 | 15 | 0 | 0 | 0 | 59 | 0,09 | 0,20 | 0,35 | 0,20 | 0,10 | 0,23 | 0,05 |
| grass | 0 | 5 | 8 | 6 | 129 | 61 | 0 | 0 | 0 | 209 | 0,19 | 0,62 | 0,47 | 0,62 | 0,07 | 0,66 | 0,04 |
| bare | 1 | 3 | 6 | 1 | 3 | 142 | 2 | 0 | 0 | 158 | 0,20 | 0,90 | 0,52 | 0,90 | 0,05 | 0,71 | 0,12 |
| water | 0 | 0 | 1 | 0 | 1 | 2 | 68 | 0 | 0 | 72 | 0,03 | 0,94 | 0,66 | 0,94 | 0,05 | 0,80 | 0,00 |
| wet | 0 | 3 | 6 | 1 | 22 | 0 | 4 | 1 | 0 | 37 | 0,00 | 0,03 | 1,00 | 0,03 | 0,05 | 1,00 | 0,00 |
| snow | 0 | 0 | 0 | 4 | 0 | 30 | 23 | 0 | 0 | 57 | 0,00 | 0,00 | 0,00 | 0,00 | 0,00 | 0,00 | 0,00 |
| Total | 35 | 118 | 619 | 34 | 275 | 273 | 103 | 1 | 0 | 1458 | 1,00 | **OAA** | 0,71 | **OAA** | 0,79 | **OAA ±** | 0,02 |

#### Table S8**.** Error matrix for ESA WC Alps, Metrics as in Table S2.

|  | **Reference** | | | | | | | | |  | **Map** | | | **Adjusted** | | | |
| --- | --- | --- | --- | --- | --- | --- | --- | --- | --- | --- | --- | --- | --- | --- | --- | --- | --- |
| **ESA WC** | built | crop | forest | shrub | grass | bare | water | wet | snow | Total | Wi | UA | PA | UA | UA ± | PA | PA ± |
| built | 41 | 0 | 18 | 1 | 12 | 9 | 0 | 0 | 0 | 81 | 0,02 | 0,51 | 0,91 | 0,51 | 0,11 | 0,79 | 0,14 |
| crop | 1 | 81 | 11 | 2 | 43 | 1 | 0 | 0 | 0 | 139 | 0,05 | 0,58 | 0,91 | 0,58 | 0,08 | 0,76 | 0,02 |
| forest | 2 | 0 | 627 | 1 | 13 | 3 | 0 | 0 | 0 | 646 | 0,50 | 0,97 | 0,87 | 0,97 | 0,01 | 0,91 | 0,13 |
| shrub | 0 | 0 | 26 | 6 | 26 | 1 | 0 | 0 | 0 | 59 | 0,03 | 0,10 | 0,55 | 0,10 | 0,08 | 0,11 | 0,03 |
| grass | 1 | 7 | 26 | 1 | 170 | 4 | 0 | 0 | 0 | 209 | 0,24 | 0,81 | 0,51 | 0,81 | 0,05 | 0,81 | 0,10 |
| bare | 0 | 0 | 6 | 0 | 49 | 85 | 0 | 0 | 18 | 158 | 0,05 | 0,54 | 0,81 | 0,54 | 0,08 | 0,76 | 0,00 |
| water | 0 | 0 | 1 | 0 | 1 | 1 | 69 | 0 | 0 | 72 | 0,02 | 0,96 | 0,96 | 0,96 | 0,05 | 1,00 | 0,00 |
| wet | 0 | 1 | 7 | 0 | 16 | 0 | 3 | 10 | 0 | 37 | 0,00 | 0,27 | 1,00 | 0,27 | 0,15 | 1,00 | 0,10 |
| snow | 0 | 0 | 0 | 0 | 1 | 1 | 0 | 0 | 55 | 57 | 0,02 | 0,75 | 0,75 | 0,96 | 0,05 | 0,67 |  |
| Total | 45 | 89 | 722 | 11 | 331 | 105 | 72 | 10 | 73 | 1458 | 0,92 | **OAA** | 0,78 | **OAA** | 0,86 | **OAA ±** | 0,02 |

#### Table S9**.** Error matrix for ESRI Alps, Metrics as in Table S2.

|  | **Reference** | | | | | | | | |  | **Map** | | | **Adjusted** | | | |
| --- | --- | --- | --- | --- | --- | --- | --- | --- | --- | --- | --- | --- | --- | --- | --- | --- | --- |
| **ESRI LC** | built | crop | forest | shrub | grass | bare | water | wet | snow | Total | Wi | UA | PA | UA | UA ± | PA | PA ± |
| built | 64 | 3 | 2 | 4 | 2 | 4 | 2 | 0 | 0 | 81 | 0,07 | 0,79 | 0,62 | 0,79 | 0,09 | 0,70 | 0,04 |
| crop | 6 | 124 | 2 | 0 | 6 | 1 | 0 | 0 | 0 | 139 | 0,13 | 0,89 | 0,68 | 0,89 | 0,05 | 0,81 | 0,03 |
| forest | 16 | 10 | 567 | 42 | 8 | 3 | 0 | 0 | 0 | 646 | 0,47 | 0,88 | 0,86 | 0,88 | 0,03 | 0,78 | 0,09 |
| shrub | 0 | 0 | 35 | 22 | 1 | 1 | 0 | 0 | 0 | 59 | 0,16 | 0,37 | 0,10 | 0,37 | 0,12 | 0,46 | 0,14 |
| grass | 14 | 38 | 43 | 54 | 55 | 1 | 4 | 0 | 0 | 209 | 0,09 | 0,26 | 0,68 | 0,26 | 0,06 | 0,57 | 0,18 |
| bare | 4 | 1 | 10 | 75 | 4 | 39 | 1 | 0 | 24 | 158 | 0,03 | 0,25 | 0,74 | 0,25 | 0,07 | 0,42 | 0,09 |
| water | 0 | 1 | 0 | 0 | 0 | 0 | 71 | 0 | 0 | 72 | 0,02 | 0,99 | 0,88 | 0,99 | 0,03 | 0,86 | 0,00 |
| wet | 0 | 5 | 4 | 17 | 5 | 1 | 3 | 2 | 0 | 37 | 0,01 | 0,05 | 1,00 | 0,05 | 0,07 | 1,00 | 0,06 |
| snow | 0 | 0 | 0 | 1 | 0 | 3 | 0 | 0 | 53 | 57 | 0,02 | 0,69 | 0,69 | 0,93 | 0,07 | 0,80 |  |
| Total | 104 | 182 | 663 | 215 | 81 | 53 | 81 | 2 | 77 | 1458 | 1,00 | **OAA** | 0,68 | **OAA** | 0,72 | **OAA ±** | 0,03 |

#### Table S10**.** Error matrix for S2GLC Alps, Metrics as in Table S2.

|  | **Reference** | | | | | | | | |  | **Map** | | | **Adjusted** | | | |
| --- | --- | --- | --- | --- | --- | --- | --- | --- | --- | --- | --- | --- | --- | --- | --- | --- | --- |
| **S2GLC** | built | crop | forest | shrub | grass | bare | water | wet | snow | Total | Wi | UA | PA | UA | UA ± | PA | PA ± |
| built | 44 | 20 | 1 | 4 | 6 | 2 | 1 | 1 | 0 | 79 | 0,02 | 0,56 | 0,86 | 0,56 | 0,11 | 0,74 | 0,06 |
| crop | 2 | 102 | 0 | 0 | 33 | 0 | 0 | 2 | 0 | 139 | 0,07 | 0,73 | 0,60 | 0,73 | 0,07 | 0,64 | 0,03 |
| forest | 0 | 14 | 510 | 67 | 21 | 0 | 0 | 18 | 0 | 630 | 0,38 | 0,81 | 0,96 | 0,81 | 0,03 | 0,92 | 0,08 |
| shrub | 0 | 3 | 7 | 23 | 9 | 1 | 0 | 5 | 0 | 48 | 0,08 | 0,48 | 0,15 | 0,48 | 0,14 | 0,36 | 0,06 |
| grass | 1 | 27 | 12 | 34 | 118 | 1 | 0 | 15 | 0 | 208 | 0,15 | 0,57 | 0,59 | 0,57 | 0,07 | 0,62 | 0,11 |
| bare | 4 | 4 | 2 | 25 | 13 | 83 | 4 | 0 | 16 | 151 | 0,05 | 0,55 | 0,93 | 0,55 | 0,08 | 0,89 | 0,07 |
| water | 0 | 0 | 0 | 0 | 0 | 0 | 71 | 1 | 0 | 72 | 0,02 | 0,99 | 0,87 | 0,99 | 0,03 | 0,86 | 0,08 |
| wet | 0 | 0 | 0 | 0 | 0 | 0 | 0 | 37 | 0 | 37 | 0,01 | 1,00 | 0,47 | 1,00 | 0,00 | 0,38 | 0,13 |
| snow | 0 | 0 | 0 | 0 | 0 | 2 | 6 | 0 | 21 | 29 | 0,01 | 0,57 | 0,57 | 0,72 | 0,17 | 0,49 |  |
| Total | 51 | 170 | 532 | 153 | 200 | 89 | 82 | 79 | 37 | 1393 | 0,79 | **OAA** | 0,72 | **OAA** | 0,70 | **OAA ±** | 0,03 |

# Supplementary material: Error Matrices - Carpathians

#### Table S11**.** Error matrix for Con_RF Carpathians, Metrics as in Table S2.

|  | **Reference** | | | | | | | | | **Map** | | | **Adjusted** | | | |
| --- | --- | --- | --- | --- | --- | --- | --- | --- | --- | --- | --- | --- | --- | --- | --- | --- |
| **Con_RF** | built | crop | forest | shrub | grass | bare | water | wet | Total | Wi | UA | PA | UA | UA ± | PA | PA ± |
| built | 65 | 0 | 6 | 0 | 3 | 0 | 0 | 0 | 74 | 0,03 | 0,88 | 0,77 | 0,88 | 0,07 | 0,71 | 0,05 |
| crop | 3 | 168 | 2 | 0 | 35 | 0 | 0 | 0 | 208 | 0,12 | 0,81 | 0,97 | 0,81 | 0,05 | 0,94 | 0,02 |
| forest | 5 | 0 | 658 | 0 | 10 | 0 | 0 | 0 | 673 | 0,57 | 0,98 | 0,86 | 0,98 | 0,01 | 0,95 | 0,21 |
| shrub | 0 | 0 | 65 | 7 | 8 | 0 | 0 | 0 | 80 | 0,05 | 0,09 | 0,78 | 0,09 | 0,06 | 0,12 | 0,03 |
| grass | 6 | 5 | 18 | 1 | 161 | 0 | 1 | 0 | 192 | 0,22 | 0,84 | 0,63 | 0,84 | 0,05 | 0,87 | 0,00 |
| bare | 5 | 0 | 4 | 0 | 15 | 29 | 7 | 0 | 60 | 0,00 | 0,48 | 1,00 | 0,48 | 0,13 | 1,00 | 0,28 |
| water | 0 | 0 | 2 | 0 | 1 | 0 | 74 | 0 | 77 | 0,01 | 0,96 | 0,88 | 0,96 | 0,04 | 0,81 | 0,00 |
| wet | 0 | 0 | 13 | 1 | 23 | 0 | 2 | 0 | 39 | 0,00 | 0,00 | 0,00 | 0,00 | 0,00 | 0,00 | 0,00 |
| Total | 84 | 173 | 768 | 9 | 256 | 29 | 84 | 0 | 1403 | 1,00 | **OAA** | 0,83 | **OAA** | 0,92 | **OAA ±** | 0,02 |

#### Table S12**.** Error matrix for Con_AccCo Carpathians, Metrics as in Table S2.

|  | **Reference** | | | | | | | | | **Map** | | | **Adjusted** | | | |
| --- | --- | --- | --- | --- | --- | --- | --- | --- | --- | --- | --- | --- | --- | --- | --- | --- |
| **Con_AccCo** | built | crop | forest | shrub | grass | bare | water | wet | Total | Wi | UA | PA | UA | UA ± | PA | PA ± |
| built | 64 | 3 | 6 | 0 | 1 | 0 | 0 | 0 | 74 | 0,03 | 0,86 | 0,80 | 0,86 | 0,08 | 0,69 | 0,05 |
| crop | 4 | 196 | 0 | 0 | 8 | 0 | 0 | 0 | 208 | 0,16 | 0,94 | 0,82 | 0,94 | 0,03 | 0,84 | 0,02 |
| forest | 5 | 2 | 660 | 0 | 5 | 0 | 1 | 0 | 673 | 0,58 | 0,98 | 0,84 | 0,98 | 0,01 | 0,95 | 0,00 |
| shrub | 0 | 1 | 73 | 1 | 5 | 0 | 0 | 0 | 80 | 0,05 | 0,01 | 1,00 | 0,01 | 0,02 | 1,00 | 0,04 |
| grass | 6 | 26 | 25 | 0 | 133 | 0 | 1 | 1 | 192 | 0,17 | 0,69 | 0,81 | 0,69 | 0,07 | 0,92 | 0,12 |
| bare | 1 | 7 | 3 | 0 | 8 | 38 | 3 | 0 | 60 | 0,00 | 0,63 | 0,97 | 0,63 | 0,12 | 0,93 | 0,25 |
| water | 0 | 0 | 1 | 0 | 0 | 1 | 75 | 0 | 77 | 0,01 | 0,97 | 0,91 | 0,97 | 0,04 | 0,75 | 0,15 |
| wet | 0 | 5 | 15 | 0 | 5 | 0 | 2 | 12 | 39 | 0,00 | 0,31 | 0,92 | 0,31 | 0,15 | 0,08 |  |
| Total | 80 | 240 | 783 | 1 | 165 | 39 | 82 | 13 | 1403 | 1,00 | **OAA** | 0,84 | **OAA** | 0,91 | **OAA ±** | 0,02 |

#### Table S13**.** Error matrix for Con_WV Carpathians, Metrics as in Table S2.

|  | **Reference** | | | | | | | | | **Map** | | | **Adjusted** | | | |
| --- | --- | --- | --- | --- | --- | --- | --- | --- | --- | --- | --- | --- | --- | --- | --- | --- |
| **Con_WV** | built | crop | forest | shrub | grass | bare | water | wet | Total | Wi | UA | PA | UA | UA ± | PA | PA ± |
| built | 61 | 6 | 6 | 0 | 1 | 0 | 0 | 0 | 74 | 0,02 | 0,82 | 0,87 | 0,82 | 0,09 | 0,76 | 0,04 |
| crop | 1 | 198 | 1 | 0 | 8 | 0 | 0 | 0 | 208 | 0,16 | 0,95 | 0,81 | 0,95 | 0,03 | 0,83 | 0,02 |
| forest | 3 | 3 | 659 | 0 | 7 | 0 | 1 | 0 | 673 | 0,58 | 0,98 | 0,84 | 0,98 | 0,01 | 0,95 | 0,00 |
| shrub | 0 | 1 | 73 | 1 | 5 | 0 | 0 | 0 | 80 | 0,05 | 0,01 | 1,00 | 0,01 | 0,02 | 1,00 | 0,04 |
| grass | 4 | 26 | 25 | 0 | 135 | 0 | 1 | 1 | 192 | 0,18 | 0,70 | 0,79 | 0,70 | 0,06 | 0,91 | 0,11 |
| bare | 1 | 5 | 3 | 0 | 8 | 40 | 3 | 0 | 60 | 0,00 | 0,67 | 0,98 | 0,67 | 0,12 | 0,94 | 0,25 |
| water | 0 | 0 | 1 | 0 | 0 | 1 | 75 | 0 | 77 | 0,01 | 0,97 | 0,90 | 0,97 | 0,04 | 0,75 | 0,06 |
| wet | 0 | 4 | 15 | 0 | 6 | 0 | 3 | 11 | 39 | 0,00 | 0,28 | 0,92 | 0,28 | 0,14 | 0,03 | 0,00 |
| Total | 70 | 243 | 783 | 1 | 170 | 41 | 83 | 12 | 1403 | 1,00 | **OAA** | 0,84 | **OAA** | 0,91 | **OAA ±** | 0,02 |

#### Table S14**.** Error matrix for CLC+ Carpathians, Metrics as in Table S2.

|  | **Reference** | | | | | | | | | **Map** | | | **Adjusted** | | | |
| --- | --- | --- | --- | --- | --- | --- | --- | --- | --- | --- | --- | --- | --- | --- | --- | --- |
| **CLC+ Backbone** | built | crop | forest | shrub | grass | bare | water | wet | Total | Wi | UA | PA | UA | UA ± | PA | PA ± |
| built | 45 | 1 | 5 | 2 | 13 | 4 | 0 | 0 | 70 | 0,01 | 0,64 | 0,94 | 0,64 | 0,11 | 0,85 | 0,06 |
| crop | 1 | 164 | 2 | 7 | 29 | 0 | 0 | 0 | 203 | 0,13 | 0,81 | 0,90 | 0,81 | 0,05 | 0,83 | 0,01 |
| forest | 2 | 0 | 540 | 18 | 18 | 2 | 0 | 0 | 580 | 0,48 | 0,93 | 0,95 | 0,93 | 0,02 | 0,97 | 0,09 |
| shrub | 0 | 0 | 13 | 45 | 13 | 0 | 0 | 0 | 71 | 0,05 | 0,63 | 0,53 | 0,63 | 0,11 | 0,39 | 0,04 |
| grass | 0 | 16 | 5 | 7 | 145 | 1 | 0 | 0 | 174 | 0,21 | 0,83 | 0,57 | 0,83 | 0,06 | 0,81 | 0,22 |
| bare | 0 | 0 | 0 | 0 | 11 | 44 | 1 | 0 | 56 | 0,00 | 0,79 | 0,85 | 0,79 | 0,11 | 0,47 | 0,03 |
| water | 0 | 1 | 1 | 0 | 1 | 1 | 72 | 0 | 76 | 0,00 | 0,95 | 0,95 | 0,95 | 0,05 | 0,98 | 0,00 |
| wet | 0 | 0 | 5 | 6 | 23 | 0 | 3 | 0 | 37 | 0,00 | 0,00 | 0,00 | 0,00 | 0,00 | 0,00 | 0,00 |
| Total | 48 | 182 | 571 | 85 | 253 | 52 | 76 | 0 | 1267 | 0,90 | **OAA** | 0,83 | **OAA** | 0,87 | **OAA ±** | 0,02 |

#### Table S15**.** Error matrix for DW Carpathians, Metrics as in Table S2.

|  | **Reference** | | | | | | | | | **Map** | | | **Adjusted** | | | |
| --- | --- | --- | --- | --- | --- | --- | --- | --- | --- | --- | --- | --- | --- | --- | --- | --- |
| **DW** | built | crop | forest | shrub | grass | bare | water | wet | Total | Wi | UA | PA | UA | UA ± | PA | PA ± |
| built | 70 | 1 | 3 | 0 | 0 | 0 | 0 | 0 | 74 | 0,05 | 0,95 | 0,68 | 0,95 | 0,05 | 0,69 | 0,04 |
| crop | 7 | 184 | 5 | 2 | 10 | 0 | 0 | 0 | 208 | 0,15 | 0,88 | 0,87 | 0,88 | 0,04 | 0,89 | 0,01 |
| forest | 12 | 3 | 654 | 0 | 2 | 0 | 2 | 0 | 673 | 0,65 | 0,97 | 0,77 | 0,97 | 0,01 | 0,91 | 0,00 |
| shrub | 0 | 1 | 78 | 0 | 1 | 0 | 0 | 0 | 80 | 0,02 | 0,00 | 0,00 | 0,00 | 0,00 | 0,00 | 0,08 |
| grass | 12 | 22 | 70 | 4 | 81 | 0 | 1 | 0 | 190 | 0,12 | 0,43 | 0,81 | 0,42 | 0,07 | 0,84 | 0,00 |
| bare | 2 | 0 | 4 | 23 | 3 | 12 | 2 | 0 | 46 | 0,00 | 0,26 | 1,00 | 0,20 | 0,10 | 1,00 | 0,23 |
| water | 0 | 0 | 1 | 0 | 0 | 0 | 76 | 0 | 77 | 0,01 | 0,99 | 0,92 | 0,99 | 0,03 | 0,71 | 0,00 |
| wet | 0 | 1 | 30 | 0 | 3 | 0 | 2 | 3 | 39 | 0,01 | 0,08 | 1,00 | 0,08 | 0,08 | 1,00 |  |
| Total | 103 | 212 | 845 | 29 | 100 | 12 | 83 | 3 | 1387 | 1,00 | **OAA** | 0,78 | **OAA** | 0,88 | **OAA ±** | 0,01 |

#### Table S16**.** Error matrix for ELC10 Carpathians, Metrics as in Table S2.

|  | **Reference** | | | | | | | | | **Map** | | | **Adjusted** | | | |
| --- | --- | --- | --- | --- | --- | --- | --- | --- | --- | --- | --- | --- | --- | --- | --- | --- |
| **ELC10** | built | crop | forest | shrub | grass | bare | water | wet | Total | Wi | UA | PA | UA | UA ± | PA | PA ± |
| built | 31 | 19 | 6 | 0 | 16 | 2 | 0 | 0 | 74 | 0,01 | 0,42 | 0,97 | 0,42 | 0,11 | 0,85 | 0,05 |
| crop | 0 | 181 | 0 | 0 | 27 | 0 | 0 | 0 | 208 | 0,14 | 0,87 | 0,69 | 0,87 | 0,05 | 0,77 | 0,02 |
| forest | 1 | 8 | 637 | 1 | 25 | 1 | 0 | 0 | 673 | 0,55 | 0,95 | 0,89 | 0,95 | 0,02 | 0,96 | 0,28 |
| shrub | 0 | 3 | 46 | 13 | 11 | 4 | 2 | 1 | 80 | 0,08 | 0,16 | 0,93 | 0,16 | 0,08 | 0,16 | 0,04 |
| grass | 0 | 25 | 16 | 0 | 143 | 7 | 0 | 1 | 192 | 0,21 | 0,74 | 0,61 | 0,74 | 0,06 | 0,80 | 0,15 |
| bare | 0 | 12 | 1 | 0 | 1 | 46 | 0 | 0 | 60 | 0,01 | 0,77 | 0,75 | 0,77 | 0,11 | 0,31 | 0,01 |
| water | 0 | 2 | 2 | 0 | 2 | 1 | 70 | 0 | 77 | 0,01 | 0,91 | 0,93 | 0,91 | 0,06 | 0,99 | 0,11 |
| forest | 0 | 12 | 10 | 0 | 8 | 0 | 3 | 6 | 39 | 0,00 | 0,15 | 0,75 | 0,15 | 0,11 | 0,06 |  |
| Total | 32 | 262 | 718 | 14 | 233 | 61 | 75 | 8 | 1403 | 1,00 | **OAA** | 0,80 | **OAA** | 0,88 | **OAA ±** | 0,02 |

#### Table S17**.** Error matrix for ESA WC Carpathians, Metrics as in Table S2.

|  | **Reference** | | | | | | | | | **Map** | | | **Adjusted** | | | |
| --- | --- | --- | --- | --- | --- | --- | --- | --- | --- | --- | --- | --- | --- | --- | --- | --- |
| **ESA WC** | built | crop | forest | shrub | grass | bare | water | wet | Total | Wi | UA | PA | UA | UA ± | PA | PA ± |
| built | 41 | 2 | 19 | 0 | 7 | 5 | 0 | 0 | 74 | 0,02 | 0,55 | 0,93 | 0,55 | 0,11 | 0,80 | 0,05 |
| crop | 0 | 183 | 3 | 0 | 21 | 1 | 0 | 0 | 208 | 0,16 | 0,88 | 0,86 | 0,88 | 0,04 | 0,84 | 0,02 |
| forest | 1 | 2 | 654 | 0 | 15 | 1 | 0 | 0 | 673 | 0,59 | 0,97 | 0,84 | 0,97 | 0,01 | 0,94 | 0,00 |
| shrub | 0 | 0 | 61 | 0 | 18 | 0 | 0 | 1 | 80 | 0,00 | 0,00 | 0,00 | 0,00 | 0,00 |  | 0,04 |
| grass | 1 | 21 | 23 | 0 | 143 | 1 | 0 | 3 | 192 | 0,22 | 0,74 | 0,57 | 0,74 | 0,06 | 0,83 | 0,14 |
| bare | 1 | 2 | 4 | 0 | 38 | 8 | 7 | 0 | 60 | 0,01 | 0,13 | 0,50 | 0,13 | 0,09 | 0,15 | 0,07 |
| water | 0 | 1 | 2 | 0 | 2 | 0 | 70 | 2 | 77 | 0,01 | 0,91 | 0,90 | 0,91 | 0,06 | 0,89 | 0,11 |
| wet | 0 | 3 | 8 | 0 | 6 | 0 | 1 | 21 | 39 | 0,00 | 0,54 | 0,78 | 0,54 | 0,16 | 0,11 |  |
| Total | 44 | 214 | 774 | 0 | 250 | 16 | 78 | 27 | 1403 | 1,00 | **OAA** | 0,80 | **OAA** | 0,90 | **OAA ±** | 0,02 |

#### Table S18**.** Error matrix for ESRI LC Carpathians, Metrics as in Table S2.

|  | **Reference** | | | | | | | | | **Map** | | | **Adjusted** | | | |
| --- | --- | --- | --- | --- | --- | --- | --- | --- | --- | --- | --- | --- | --- | --- | --- | --- |
| **ESRI LC** | built | crop | forest | shrub | grass | bare | water | wet | Total | Wi | UA | PA | UA | UA ± | PA | PA ± |
| built | 69 | 1 | 3 | 1 | 0 | 0 | 0 | 0 | 74 | 0,06 | 0,93 | 0,60 | 0,93 | 0,06 | 0,67 | 0,03 |
| crop | 7 | 194 | 0 | 3 | 4 | 0 | 0 | 0 | 208 | 0,21 | 0,93 | 0,65 | 0,93 | 0,03 | 0,78 | 0,01 |
| forest | 18 | 23 | 576 | 34 | 17 | 0 | 3 | 2 | 673 | 0,53 | 0,86 | 0,86 | 0,86 | 0,03 | 0,87 | 0,07 |
| shrub | 0 | 7 | 62 | 5 | 6 | 0 | 0 | 0 | 80 | 0,06 | 0,06 | 0,00 | 0,06 | 0,05 | 0,08 | 0,10 |
| grass | 17 | 53 | 24 | 25 | 72 | 0 | 1 | 0 | 192 | 0,11 | 0,38 | 0,71 | 0,37 | 0,07 | 0,64 | 0,52 |
| bare | 2 | 6 | 1 | 40 | 1 | 8 | 2 | 0 | 60 | 0,00 | 0,13 | 0,89 | 0,13 | 0,09 | 0,48 | 0,21 |
| water | 2 | 1 | 1 | 1 | 0 | 1 | 71 | 0 | 77 | 0,01 | 0,92 | 0,84 | 0,92 | 0,06 | 0,67 | 0,04 |
| wet | 0 | 14 | 6 | 1 | 1 | 0 | 8 | 9 | 39 | 0,01 | 0,23 | 0,82 | 0,23 | 0,13 | 0,03 |  |
| Total | 115 | 299 | 673 | 110 | 101 | 9 | 85 | 11 | 1403 | 1,00 | **OAA** | 0,72 | **OAA** | 0,77 | **OAA ±** | 0,02 |

#### Table S19**.** Error matrix for S2GLC Carpathians, Metrics as in Table S2.

|  | **Reference** | | | | | | | | | **Map** | | | **Adjusted** | | | |
| --- | --- | --- | --- | --- | --- | --- | --- | --- | --- | --- | --- | --- | --- | --- | --- | --- |
| **S2GLC** | built | crop | forest | shrub | grass | bare | water | wet | Total | Wi | UA | PA | UA | UA ± | PA | PA ± |
| built | 32 | 29 | 1 | 2 | 3 | 2 | 1 | 4 | 74 | 0,06 | 0,43 | 0,76 | 0,43 | 0,11 | 0,68 | 0,04 |
| crop | 0 | 182 | 0 | 0 | 20 | 0 | 0 | 6 | 208 | 0,21 | 0,88 | 0,67 | 0,88 | 0,05 | 0,75 | 0,01 |
| forest | 1 | 18 | 586 | 8 | 33 | 0 | 1 | 26 | 673 | 0,53 | 0,87 | 0,95 | 0,87 | 0,03 | 0,97 | 0,13 |
| shrub | 0 | 5 | 19 | 43 | 7 | 0 | 0 | 6 | 80 | 0,06 | 0,54 | 0,00 | 0,54 | 0,11 | 0,40 | 0,05 |
| grass | 1 | 34 | 6 | 5 | 127 | 1 | 1 | 17 | 192 | 0,11 | 0,66 | 0,66 | 0,66 | 0,07 | 0,75 | 0,36 |
| bare | 7 | 5 | 0 | 14 | 0 | 29 | 1 | 2 | 58 | 0,00 | 0,50 | 0,85 | 0,50 | 0,13 | 0,50 | 0,28 |
| water | 1 | 0 | 2 | 1 | 0 | 2 | 66 | 5 | 77 | 0,01 | 0,86 | 0,94 | 0,86 | 0,08 | 0,68 | 0,07 |
| wet | 0 | 0 | 2 | 0 | 1 | 0 | 0 | 36 | 39 | 0,01 | 0,92 | 0,35 | 0,92 | 0,08 | 0,41 |  |
| Total | 42 | 273 | 616 | 73 | 191 | 34 | 70 | 102 | 1401 | 1,00 | **OAA** | 0,79 | **OAA** | 0,82 | **OAA ±** | 0,02 |

# References

[1.](https://www.zotero.org/google-docs/?0yj3WL) Squires, V. R., Dengler, J., Feng, H., & Hua, L. (2018). *Grasslands of the World*. CRC Press.

[2.](https://www.zotero.org/google-docs/?0yj3WL) [Bezák, P. & Mitchley, J. Drivers of change in mountain farming in Slovakia: from socialist collectivisation to the Common Agricultural Policy. *Reg. Environ. Change* **14**, 1343–1356 (2014).](https://www.zotero.org/google-docs/?0yj3WL)

[3.](https://www.zotero.org/google-docs/?0yj3WL) [Brown, C. F. *et al.* Dynamic World, Near real-time global 10 m land use land cover mapping. *Sci. Data* **9**, 251 (2022).](https://www.zotero.org/google-docs/?0yj3WL)

[4.](https://www.zotero.org/google-docs/?0yj3WL) [Karra, K. *et al.* Global land use / land cover with Sentinel 2 and deep learning. in *2021 IEEE International Geoscience and Remote Sensing Symposium IGARSS* 4704–4707 (2021). doi:10.1109/IGARSS47720.2021.9553499.](https://www.zotero.org/google-docs/?0yj3WL)

[5.](https://www.zotero.org/google-docs/?0yj3WL) [Zanaga, D. *et al.* ESA WorldCover 10 m 2020 v100. Zenodo https://doi.org/10.5281/ZENODO.5571936 (2021).](https://www.zotero.org/google-docs/?0yj3WL)

[6.](https://www.zotero.org/google-docs/?0yj3WL) [Venter, Z. S. & Sydenham, M. A. K. Continental-Scale Land Cover Mapping at 10 m Resolution Over Europe (ELC10). *Remote Sens.* **13**, 2301 (2021).](https://www.zotero.org/google-docs/?0yj3WL)

[7.](https://www.zotero.org/google-docs/?0yj3WL) [Malinowski, R. *et al.* Automated Production of a Land Cover/Use Map of Europe Based on Sentinel-2 Imagery. *Remote Sens.* **12**, 3523 (2020).](https://www.zotero.org/google-docs/?0yj3WL)

[8.](https://www.zotero.org/google-docs/?0yj3WL) [Breiman, L. Random Forest. *Mach. Learn.* **45**, 5–32 (2001).](https://www.zotero.org/google-docs/?0yj3WL)

[9.](https://www.zotero.org/google-docs/?0yj3WL) [Belgiu, M. & Drăguţ, L. Random forest in remote sensing: A review of applications and future directions. *ISPRS J. Photogramm. Remote Sens.* **114**, 24–31 (2016).](https://www.zotero.org/google-docs/?0yj3WL)

[10.](https://www.zotero.org/google-docs/?0yj3WL) [d’Andrimont, R. *et al.* Harmonised LUCAS in-situ land cover and use database for field surveys from 2006 to 2018 in the European Union. *Sci. Data* **7**, 352 (2020).](https://www.zotero.org/google-docs/?0yj3WL)

[11.](https://www.zotero.org/google-docs/?0yj3WL) [d’Andrimont, R. *et al.* LUCAS Copernicus 2018: Earth-observation-relevant in situ data on land cover and use throughout the European Union. *Earth Syst. Sci. Data* **13**, 1119–1133 (2021).](https://www.zotero.org/google-docs/?0yj3WL)

[12.](https://www.zotero.org/google-docs/?0yj3WL) [Tsendbazar, N. E., De Bruin, S., Mora, B., Schouten, L. & Herold, M. Comparative assessment of thematic accuracy of GLC maps for specific applications using existing reference data. *Int. J. Appl. Earth Obs. Geoinformation* **44**, 124–135 (2016).](https://www.zotero.org/google-docs/?0yj3WL)

[13.](https://www.zotero.org/google-docs/?0yj3WL) [Tuanmu, M. & Jetz, W. A global 1‐km consensus land‐cover product for biodiversity and ecosystem modelling. *Glob. Ecol. Biogeogr.* **23**, 1031–1045 (2014).](https://www.zotero.org/google-docs/?0yj3WL)

[14.](https://www.zotero.org/google-docs/?0yj3WL) [Olofsson, P. *et al.* Good practices for estimating area and assessing accuracy of land change. *Remote Sens. Environ.* **148**, 42–57 (2014).](https://www.zotero.org/google-docs/?0yj3WL)
